# Linkage and association mapping reveals the genetic basis of brown fibre (*Gossypium hirsutum*)

**DOI:** 10.1111/pbi.12902

**Published:** 2018-03-24

**Authors:** Tianwang Wen, Mi Wu, Chao Shen, Bin Gao, De Zhu, Xianlong Zhang, Chunyuan You, Zhongxu Lin

**Affiliations:** ^1^ National Key Laboratory of Crop Genetic Improvement College of Plant Science and Technology Huazhong Agricultural University Wuhan China; ^2^ Cotton Research Institute Shihezi Academy of Agriculture Science Shihezi Xinjiang China

**Keywords:** cotton, brown fibre, introgression signature, genome‐wide association study, fibre yield, fibre quality

## Abstract

Brown fibre cotton is an environmental‐friendly resource that plays a key role in the textile industry. However, the fibre quality and yield of natural brown cotton are poor, and fundamental research on brown cotton is relatively scarce. To understand the genetic basis of brown fibre cotton, we constructed linkage and association populations to systematically examine brown fibre accessions. We fine‐mapped the brown fibre region, *Lc*_*1*_, and dissected it into 2 loci, *qBF‐A07‐1* and *qBF‐A07‐2*. The *qBF‐A07‐1* locus mediates the initiation of brown fibre production, whereas the shade of the brown fibre is affected by the interaction between *qBF‐A07‐1* and *qBF‐A07‐2. Gh_A07G2341* and *Gh_A07G0100* were identified as candidate genes for *qBF‐A07‐1* and *qBF‐A07‐2*, respectively. Haploid analysis of the signals significantly associated with these two loci showed that most tetraploid modern brown cotton accessions exhibit the introgression signature of *Gossypium barbadense*. We identified 10 quantitative trait loci (QTLs) for fibre yield and 19 QTLs for fibre quality through a genome‐wide association study (GWAS) and found that *qBF‐A07‐2* negatively affects fibre yield and quality through an epistatic interaction with *qBF‐A07‐1*. This study sheds light on the genetics of fibre colour and lint‐related traits in brown fibre cotton, which will guide the elite cultivars breeding of brown fibre cotton.

## Introduction

Cotton has been cultivated and domesticated for over 3000 years (Lee and Fang, 2015), and various aspects of fibre quality and yield have been domesticated and improved compared with the ancestral varieties (Fang *et al*., 2017). As a visible trait, fibre colour is much more accessible for selection during domestication. Naturally coloured cotton serves as an environmental‐friendly resource for human society because it does not require dyeing (Khatri *et al*., 2015). Brown and green fibre cottons are two dominant types of naturally coloured cotton (Dutt *et al*., 2004). Natural brown cotton exhibits a widespread origin; this type of fibre has been found in several species, including the following diploids and tetraploids: *Gossypium arboreum*,* G. herbaceum*,* G. barbadense*,* G. hirsutum* and *G. tomentosum* (Carvalho *et al*., 2014; Hutchinson, 1946; Murthy, 2001; Ware, 1932). However, the natural brown cotton textile market is not promising due to its low yield and quality (Feng *et al*., 2011). Thus, it is urgent to study the genetic basis of natural brown cotton to support the breeding of this environmental‐friendly resource.

Natural brown fibre represents an excellent model for the cultivation of elite coloured cotton. The genetic loci of brown fibre have been demonstrated to be *Lc*_*1*_, *Lc*_*2*_, *Lc*_*3*_, *Lc*_*4*_, *Lc*_*5*_ and *Lc*_*6*_ (Kohel, 1985); among them, *Lc*_*1*_ is the best studied one (Hinchliffe *et al*., 2016; Li *et al*., 2012; Wang *et al*., 2014). *Lc*_*1*_ is located on chromosome A07 near the telomere (Hinchliffe *et al*., 2016). It has been reported that the metabolites of proanthocyanidins occur as pigments in the seed coats of plants and are the main product related to the pigmentation of brown fibre (Feng *et al*., 2014; Lepiniec *et al*., 2006). The metabolites of proanthocyanidins belong to products of the flavonoid pathway, which is the best‐established pathway in plants. Abundant structural genes (e.g. *CHS*,* CHI*,* F3H*,* F3′5′H*,* DFR* and *ANS*) of the flavonoid pathway have been discovered in diverse of plant species (Koes *et al*., 2005). Transcription factors (TFs) have also been regarded to play a regulatory role in the flavonoid pathway (Gates *et al*., 2018; Padmaja *et al*., 2014). It has been reported that the structural genes of the flavonoid pathway are significantly up‐regulated in brown fibre (Feng *et al*., 2013, 2014, 2015; Tan *et al*., 2013).

Following the publication of several draft genomes of cotton (Li *et al*., 2014, 2015; Wang *et al*., 2012; Yuan *et al*., 2015; Zhang *et al*., 2015)_,_ the whole‐genome resequencing (WGR) approach has greatly facilitated cotton research via the genotyping of natural populations (Fang *et al*., 2017; Scheben *et al*., 2017; Wang *et al*., 2017). Linkage mapping supports the precise study of genes but is time‐ and labour‐consuming. Alternatively, association mapping identifies many more historical recombination events and shows a higher resolution, and as a result, this approach complements to the disadvantages of linkage mapping. Furthermore, this strategy allows the identification of many new loci in the complex genetic background of crops (Li *et al*., 2016a; Motamayor *et al*., 2013). Hence, it is preferable to perform combined analyses of multiple populations to dissect the genetic basis of a trait (Lee *et al*., 2014; Mahuku *et al*., 2016; Motte *et al*., 2014; Sun *et al*., 2016; Wu *et al*., 2016).

Here, we combined linkage and association analyses to systematically elucidate the genetic basis of brown fibre. In this study, we aimed to (i) fine‐map the QTLs controlling brown fibre using linkage and association populations; (ii) study the genetic structure and relationships in a panel of accessions including 100 brown fibre and 109 white fibre accessions; and (iii) exploit the relationship between brown fibre and other fibre traits and the QTLs affecting yield and quality in the brown fibre background.

## Results

### Linkage mapping of *Lc*_*1*_

As shown in the linkage population accessed by Handan208 (HD208) and the *Youse* (*ys*) brown fibre mutant, brown fibre is a particularly obvious phenotype (Figure S1). A genetic analysis of the F_2_ population revealed a 1 : 2 : 1 segregation ratio (white:light brown:dark brown) (Table S1). Therefore, an incompletely dominant locus, designated as *Lc*_*1*_ according to the published reports (Hinchliffe *et al*., 2016; Kohel, 1985), controls the phenotype of dark brown fibre.

**Figure 1:**
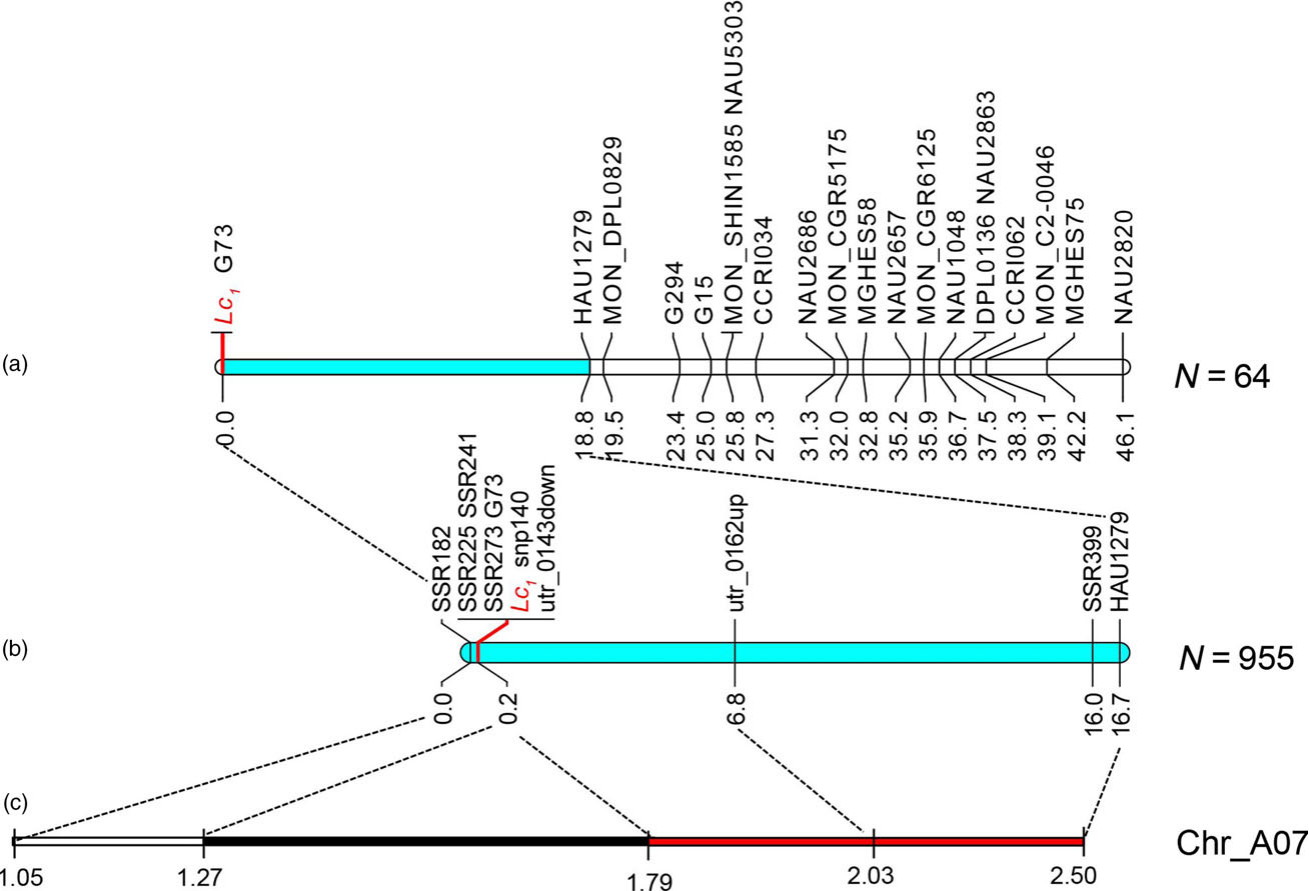
Genetic and physical map of *Lc*_*1*_. (a) Draft genetic map of *Lc*_*1*_. (b) Fine genetic map of *Lc*_*1*_. (c) Physical map of *Lc*_*1*_. The black part of the bar shows the cosegregating region, the red part of the bar shows the region of recombination hotspot and *Lc*_*1*_ represents the dark brown locus.

Based on a high‐density genetic map developed in our laboratory (Li *et al*., 2016a), polymorphic markers were screened to genotype 64 recessive individuals from 243 F_2_ plants in 2015; 17 simple sequence repeat (SSR) markers showed loose linkage with *Lc*_*1*_, and one marker cosegregated with *Lc*_*1*_ (Figure 1a). According to the draft genetic map of *Lc*_*1*_, more SSR and untranslated region (UTR) markers were developed to genotype additional 955 recessive individuals in 2016 (Figure 1b). We fine‐mapped *Lc*_*1*_ to a small region from 1.05 to 2.03 Mb on chromosome A07, and a recombination hotspot region was identified near *Lc*_*1*_; simultaneously, a long distance of cosegregating region from 1.27 to 1.79 Mb reduced the fine‐mapping resolution (Figure 1c and Table S2).

### Population structure of brown fibre accessions

**Figure 2:**
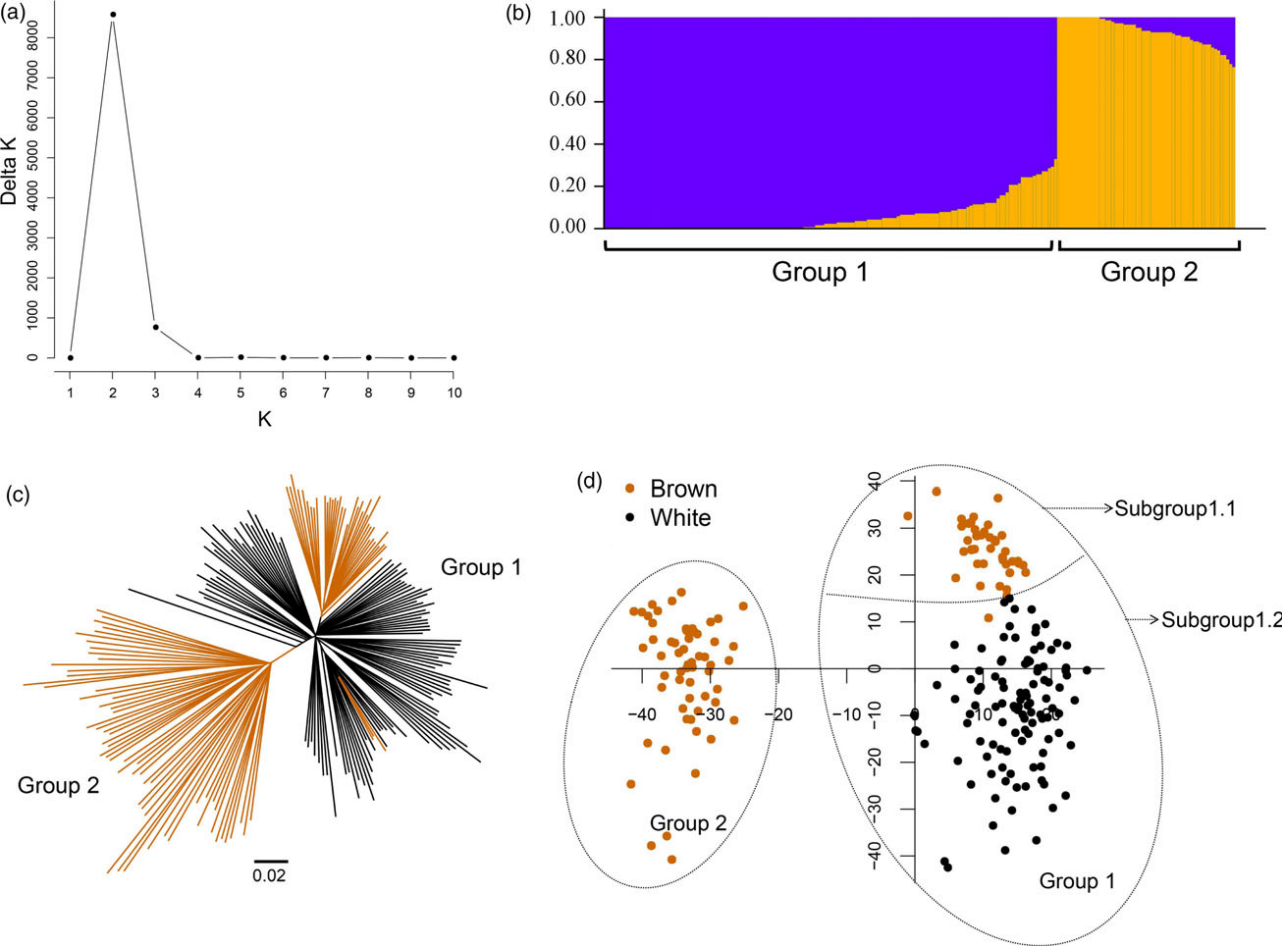
Population structure of the 209‐accession panel. (a) Delta *K* values plotted from 1 to 10. (b) Population structure of 209‐accession panel (*K *=* *2). (c) Phylogenetic tree based on Nei's genetic distance. (d) Principal component analysis of the 209‐accession panel. Brown indicates the brown fibre accessions, and black indicates white fibre accessions.

A total of 100 resequenced brown fibre cotton accessions (*G. hirsutum*) (Figure S2 and Table S3) and 109 resequenced white fibre cotton accessions (*G. hirsutum*) (Table S4) were combined to construct a panel to study their genetic structure. A population structure analysis showed that the value of Evanno's Δ*K* presented a sharp spike at *K *=* *2, which suggested that this population panel was clustered into 2 groups (Figure 2a,b). Furthermore, the results from a principal component analysis (PCA) and a phylogenetic tree agreed with the structure analysis results. The 100 brown fibre cotton accessions were distributed in two groups, with 41 and 59 accessions in Groups 1 and 2, respectively (Figure 2c). According to the PCA figure, Group 1 included both brown and white fibre accessions, but there was a clear boundary between them. Therefore, Group 1 was divided into two subgroups: Subgroup 1.1 and Subgroup 1.2. (Figure 2d and Table S5). In Subgroup 1.1, four brown fibre accessions, Z87, Z84, Z61 and Z54, were distributed around the border between Subgroups 1.1 and 1.2. All four accessions are domesticated cultivars and had undergone a clear domestication involving backcrossing with white fibre cotton (Table S3).

### Case–control association mapping of the brown fibre population

Case–control analysis of large samples is an effective method for dissecting the locus controlling brown fibre as a qualitative trait. In this study, the 100 brown fibre and 109 white fibre cotton accessions were set as the case and control groups, respectively. A total of 1 855 236 SNPs were obtained after SNP quality filtering. Based on the top 10 significant SNPs, the threshold was set to *P *<* *9.214 × e^−27^.

**Figure 3:**
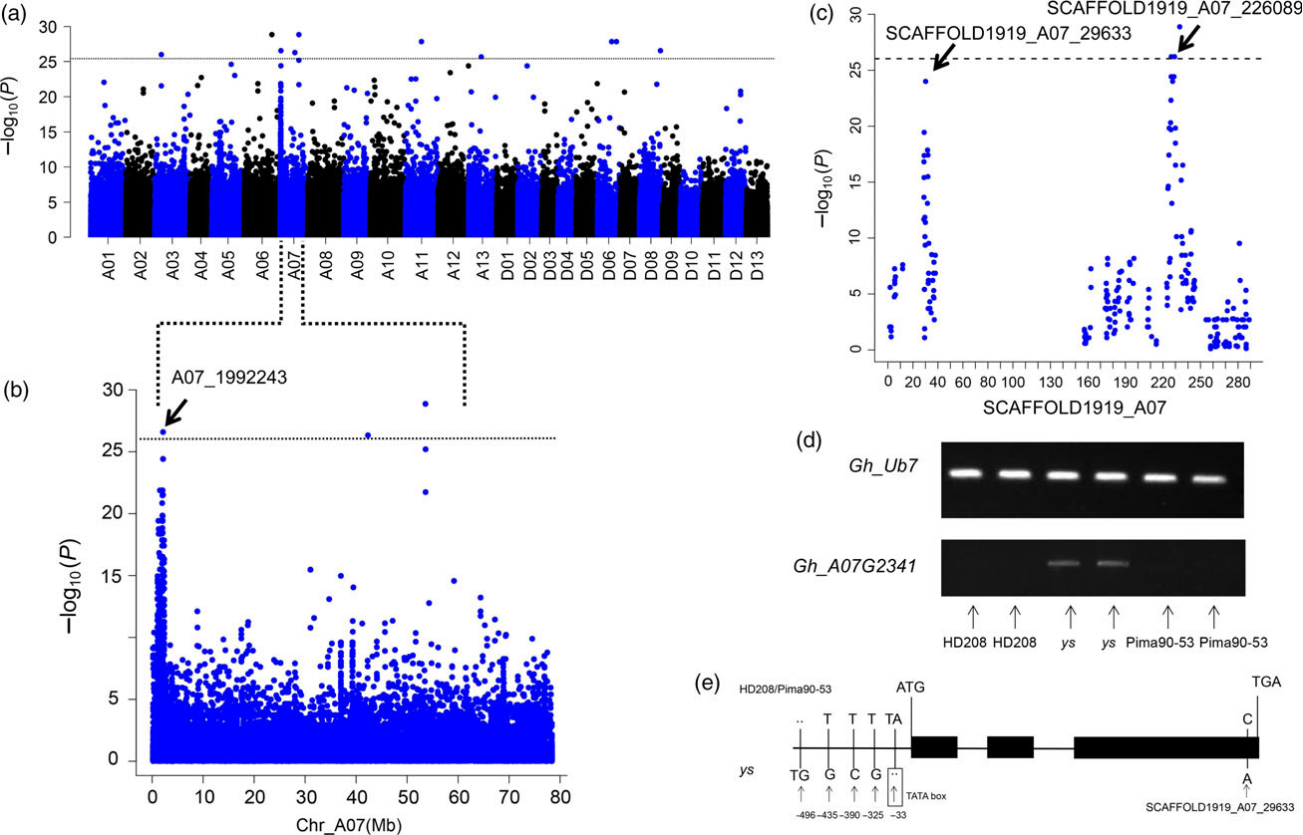
Case–control association with 209 accessions. (a) Manhattan plot with the case–control association analysis (*P *<* *9.214e^−27^). (b) Significant association signals on chromosome A07. (c) Plots of the SCAFFOLD1919_A07. The arrows indicate the significantly associated SNPs in (b) and (c). (d) Expression levels of *Gh_A07G2341* in HD208, *ys* and Pima90‐53, with *Gh_Ub7* as the control. (e) Polymorphisms of *Gh_A07G2341* among HD208, *ys* and Pima90‐53. The transcription start site (TSS) is set as the ‘0’ location in *Gh_A07G2341*.

Because the difference between the genetic backgrounds of brown and white fibres was notable, a number of significantly associated SNPs were randomly distributed across the 26 chromosomes (Figure 3a). At the telomere of chromosome A07, a continuous peak appeared from 1.0 to 2.3 Mb, which was consistent with the linkage mapping results. Further analysis of this region indicated that the SNP A07_1992243 was at the top of this peak (Figure 3b). Notably, significantly associated signals also appeared on SCAFFOLD1919_A07 (Figure 3c); according to the collinear relationship between the At subgenome of *G. hirsutum* and *G. arboreum*, SCAFFOLD1919_A07 was anchored to chromosome A07 (2.0–2.2 Mb).

Three SNPs around A07_1992243 as *Hap1* and two SNPs around SCAFFOLD1919_A07_226089 as *Hap2* were combined to conduct a haploid analysis; the results showed that the white fibre accessions harboured a completely different haploid type compared with the brown fibre accessions. BLAST analysis of sequences including *Hap1* and *Hap2* in the *G. barbadense* databank (https://www.cottongen.org) showed that the predominant haploid type of brown fibre originated from *G. barbadense* (Tables S6 and S7), and clone sequences from *G. barbadense* acc. Pima90‐53, *G. hirsutum* cv. HD208 and the *ys* mutant confirmed this finding (Figure S3). Based on the linkage disequilibrium (LD) value for chromosome A07 (LD = 220 kb), A07: 1.77–2.22 Mb was set as the candidate region and this major QTL was defined as *qBF‐A07‐1*. The expression levels of genes of this region were examined between Pima90‐53, HD208 and the *ys* mutant. Finally, *Gh_A07G2341* was confirmed to be significantly up‐regulated in the *ys* mutant (Figure 3d). Abundant polymorphic SNPs were found in the promoter of *Gh_A07G2341* between Pima90‐53, HD208 and *ys*. Particularly, according to the DNA element prediction website (http://bioinformatics.psb.ugent.be/webtools/plantcare/html/), an indel appeared in the TATA box DNA element, which is the core element of the promoter. Notably, the significantly associated SNP of SCAFFOLD1919_A07_29633 (C/A) was mutated in the third exon of *Gh_A07G2341*, which caused an amino acid substitution from proline to threonine (Figure 3c,e). In the association populations, abundant polymorphisms of *Gh_A07G2341* were also observed between the brown and white fibre accessions (Table S8). These results indicated that the mutated SNPs in *Gh_A07G2341* might be caused by distant hybridization between *G. barbadense* and *G. hirsutum*, thus inducing a gain‐of‐function. Gene annotation showed that *Gh_A07G2341* belongs to MYB transcription factors.

### Genome‐wide association mapping of the shade index of brown fibre

**Figure 4:**
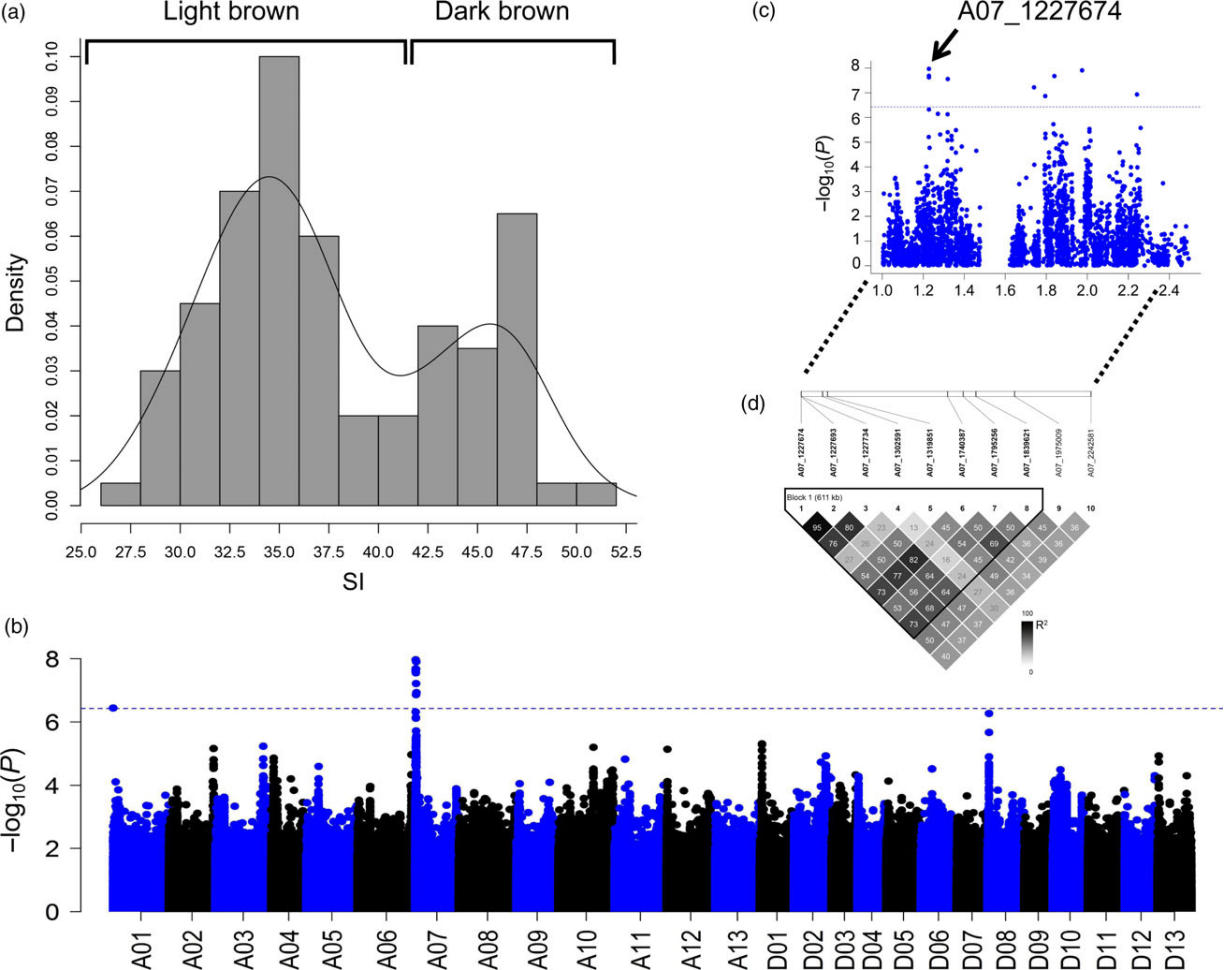
Association analysis of the shade index (SI). (a) Histogram of the SI of 100 brown fibre accessions. (b) Manhattan plot of genome‐wide associations of SI. (c) Candidate region from 1.0 to 2.5 Mb on chromosome A07. The arrow indicates the significantly associated SNP. (d) Haplotype of the candidate region constructed based on significant SNPs.

To explore and fine‐map the QTLs in the brown fibre background, 21 representative white fibre accessions were selected for combined analysis with the 100 brown accessions. A total of 2 924 715 nonredundant SNPs with a minor‐allele frequency (MAF) ≥ 0.05 were identified, resulting in 1.5 SNPs/kb. To evaluate the mapping resolution, we calculated the LD rate of this association population (Table S9). Analysis of variance (ANOVA) of the shade index (SI) of brown fibre showed notable differences between the brown fibre accessions (*F *=* *99, *P *<* *0.001) and between three environments (*F *=* *2, *P *<* *0.001), but no significant difference was observed between the replicates (*F *=* *2, *P *=* *0.6367), the heritability of SI is 0.90 calculated by 100 brown fibre accessions in multiple environments (Table S10). There were two peaks in the SI frequency histogram; we defined 30 brown fibre accessions (SI > 42) as dark brown and 70 accessions (SI < 42) as light brown (Figure 4a and Table S3).

We performed a trait‐SNP association analysis of the 121 accessions by applying a mixed linear model (MLM) (P+Q+K) with phenotypic data from three environments and a best linear unbiased prediction (BLUP) data. The SI data for the 21 white fibre cotton accessions were input as missing values to optimize the association model. In addition, a general linear model (GLM) was also applied, and the results were mostly consistent with the MLM (Figures S4–S9). Finally, the MLM was adopted to compare the QTLs of other traits under the uniform model. Manhattan plots showed nine significant SNPs at A07: 1.22–2.24 Mb with the BLUP data at *P *<* *3.70 × e^−7^ (*P *=* *1/2 697 228; −log_10_ *P* = 6.43) (Figure 4b). In particular, strong association signals appeared on SCAFFOLD1921_A07. According to the collinear relationship between the At subgenome of *G. hirsutum* and *G. arboreum*, SCAFFOLD1921_A07 is anchored to chromosome A07: 1.7–1.8 Mb. Based on the BLUP association signals, a region (A07: 1.22–2.23 Mb) including SCAFFOLD1921_A07 was fine‐mapped as the candidate region (Figure 4c). A haploid analysis of the significant SNPs showed that the region from 1.22 to 1.83 Mb constituted the haploid block (Figure 4d). According to the two peaks that appeared in the histogram of SI (Figure 4a) and the nonoverlapping significant association signals between this haploid block and *qBF‐A07‐1*, we defined this QTL as *qBF‐A07‐2*, which can significantly affect the SI of brown fibre.

Further analysis of the SNPs and SI revealed that two significant SNPs, A07_1227674 and SCAFFOLD1921_A07_53199, which belong to the haploid block, significantly affected the shade of brown colour at *P *<* *0.01 (Figure S10). We combined two SNPs around A07_1227674 as *Hap3* and two SNPs around SCAFFOLD1921_A07_53199 as *Hap4* to conduct a haploid analysis in the 121 accessions (Tables S11 and S12). The results showed that both Haploid1 and Haploid2 in *Hap3* and *Hap4* existed in the brown fibre accessions, whereas the white fibre accessions only harboured Haploid1. The SI value indicated that Haploid2 represented dark brown fibre accessions, whereas Haploid1 represented light brown or white fibre accessions. A BLAST analysis using the *G. barbadense* sequence (https://www.cottongen.org) showed that Haploid2 of *Hap3* and *Hap4* had introgression from *G. barbadense* (Tables S11 and S12). In general, 22 of the 30 dark brown accessions (SI > 42) could be explained by the presence of Haploid2 from *G. barbadense*; thus, a gene introgressed from *G. barbadense* might affect the SI of brown fibre. According to the SNP and functional annotations of genes, the candidate gene, *Gh_A07G100*, was annotated as a member of the WD40 family, and the significantly associated SNP of A07_1227674 in *Gh_A07G100* (Figure 4c) caused an amino acid mutation from isoleucine to threonine, which might result in functional alteration of the protein.

Based on the genome‐wide association and haploid analyses, the *Lc*_*1*_ region includes two QTLs, *qBF‐A07‐1* and *qBF‐A07‐2*. According to these two QTLs, we classified the accessions into four types (Table S13). The Type1 and Type2 accessions showed a significant difference in brown colour because they harboured different haplotypes of *qBF‐A07‐2*, whereas Type4 belonged to white fibre accessions, which only harboured a positive function of *qBF‐A07‐2* haplotype, without a functional *qBF‐A07‐1* locus. The results for the dark brown mutant in the linkage population were consistent with the epistatic analysis. From these results, we can deduce that *qBF‐A07‐1* (A07: 1.77–2.22 Mb) controls the induction of fibre colour, whereas *qBF‐A07‐2* (A07: 1.22–1.83 Mb) only affects the shade of brown colour and requires the presence of functional *qBF‐A07‐1*.

### Validation of *qBF‐A07‐1* and *qBF‐A07‐2* through genome‐wide selection signature analysis

**Figure 5:**
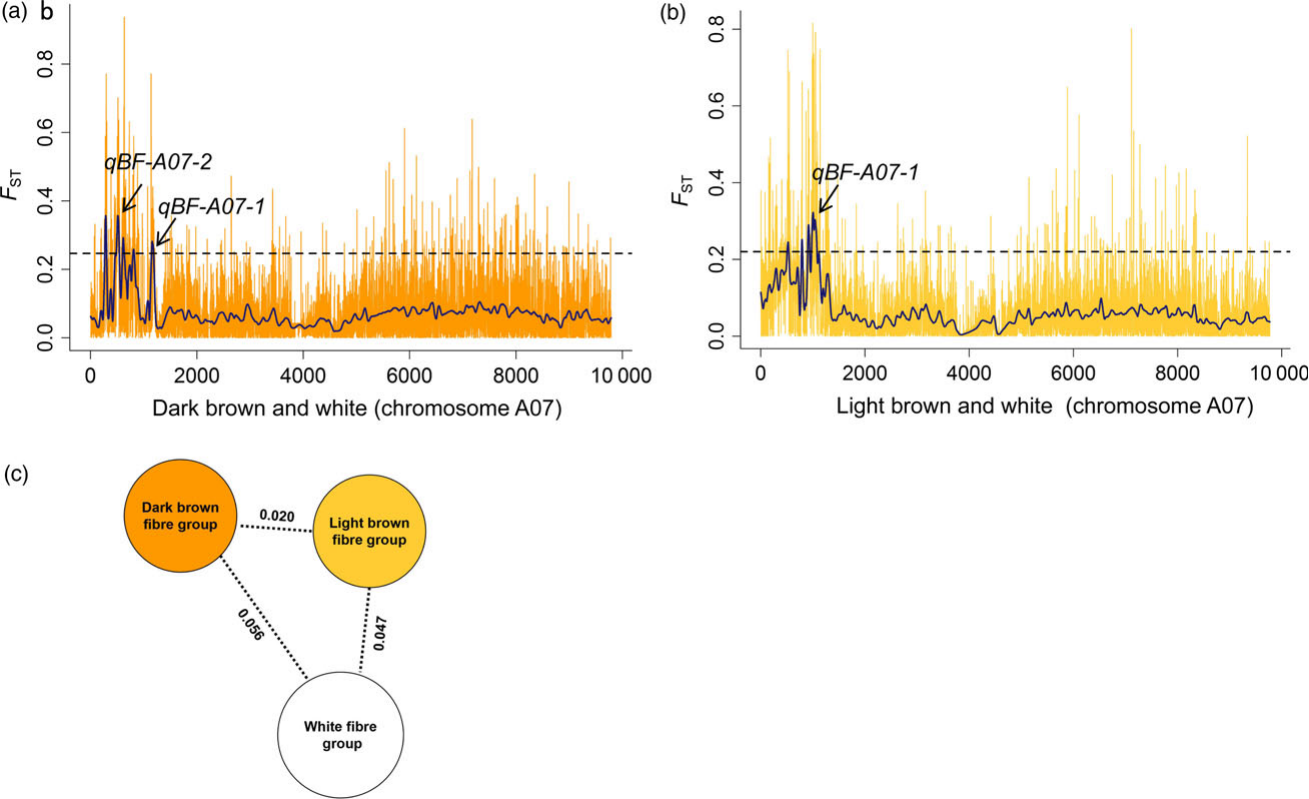
Population selection signature analysis across the dark brown, light brown and white fibre groups. (a) *F*_ST_ analysis for chromosome A07 between the dark brown fibre group (*n *=* *30) and the white fibre group (*n *=* *109). The arrows indicate the *qBF‐A07‐1* and *qBF‐A07‐2* loci. (b) Population selection signature (*F*_ST_) analysis for chromosome A07 between the light brown fibre group (*n *=* *70) and the white fibre group (*n *=* *109). The arrow indicates the *qBF‐A07‐1* locus. (c) *F*_ST_ divergence across the dark brown, light brown and white fibre groups. The value on each line indicates the population divergence value between groups.

The loci controlling brown fibre appear to have undergone selection during breeding and domestication. To validate *qBF‐A07‐1* and *qBF‐A07‐2*, a whole‐genome selection signature analysis was performed between the dark brown, light brown and white fibre accessions. Between the dark brown fibre group (*n *=* *30) and the white fibre group (*n *=* *109), the *F*_ST_ value was 0.056, suggesting a moderate differentiation between these two groups. Two peaks were identified in the region of chromosome A07 (1.06–2.03 Mb), which were consistent with the *qBF‐A07‐1* and *qBF‐A07‐2* regions. The SNP with the highest *F*_ST_ value (*F*_ST_ = 0.937) was A07_1232072 (Figure 5a,c and Table S14). Between the light brown fibre group (*n *=* *70) and the white fibre group (*n *=* *109), the *F*_ST_ value was 0.047, suggesting a weak differentiation between these two groups. A peak was found from A07_1738427 to A07_2033514 (A07: 1.73–2.03 Mb), which was consistent with the *qBF‐A07‐1* region; the SNP with the highest *F*_ST_ value (*F*_ST_ = 0.815) was A07_1865277 (Figure 5b,c and Table S15).

For the selection signature between the dark and light brown groups, the *F*_ST_ value was 0.020 (Figure 5c), exhibiting a small differentiation. The peaks on chromosome A07 showed that *qBF‐A07‐1* existed in both the dark brown and light brown fibre groups, but *qBF‐A07‐2* alone was only present in the dark brown fibre group. These results indicated that light brown accessions might have been domesticated from the dark brown accessions by discarding *qBF‐A07‐2* to achieve a better yield or quality. According to the pedigrees, Z91, Z95 and Z96 have the same brown parent, Zong9802; however, Z91 is dark brown, whereas the other accessions are light brown (Table S3). This finding indicated that domestication is useful for changing fibre colour and related traits. In a comparison of the agronomic traits, the dark brown group showed lower value than the light brown group for the fibre traits of the fibre upper half mean length (FL), fibre uniformity (FU), fibre strength (FS) and the seed cotton weight (SCW) as well as the yield traits of lint percentage (LP) and the lint weight (LW), whereas the dark brown group displayed higher values than the light brown group for the fibre traits of short fibre (SF) and fibre elongation (FE); and these differences reached the level of significance (*P *<* *0.05, two‐tailed *t*‐test). Between the light brown and white fibre groups, the light brown group exhibited lower values than the white fibre group for FL, FE, SCW, LP and LW and a higher value for SF; these differences were also significant (*P *<* *0.05, two‐tailed *t*‐test; Figure S11).

### Association mapping of fibre quality and yield traits

Brown fibre accessions exhibit a poorer yield and quality than white fibre accessions, and it is therefore necessary to exploit favourable alleles for brown cotton. The BLUP values for nine fibre yield and quality traits showed that heritability ranged from 0.76 (SCW) to 0.96 (FL), which indicated that the SCW is easily affected by the environment, whereas the FL is relatively stable. The coefficient of variation (CV) ranged from 1.57 to 31.61; the SF varied greatly between accessions, whereas the FU varied less (Table S16).

According to the population structure of the 121 accessions, the BLUP data and individual environment phenotypic data were applied to conduct association mapping by MLM (P+Q+K). The significant SNPs were annotated with snpEff (Cingolani *et al*., 2012), and QTLs were assumed based on the LD decay on the respective chromosome. In total, 19 QTLs for fibre quality and 10 QTLs for fibre yield were identified (Figures S12–S25; Table S17). Compared with the published GWAS results for cotton (*G. hirsutum*) (Huang *et al*., 2017; Sun *et al*., 2017; Wang *et al*., 2017), many new QTLs responsible for fibre quality and yield were discovered in this new panel including 100 brown fibre accessions.

**Figure 6:**
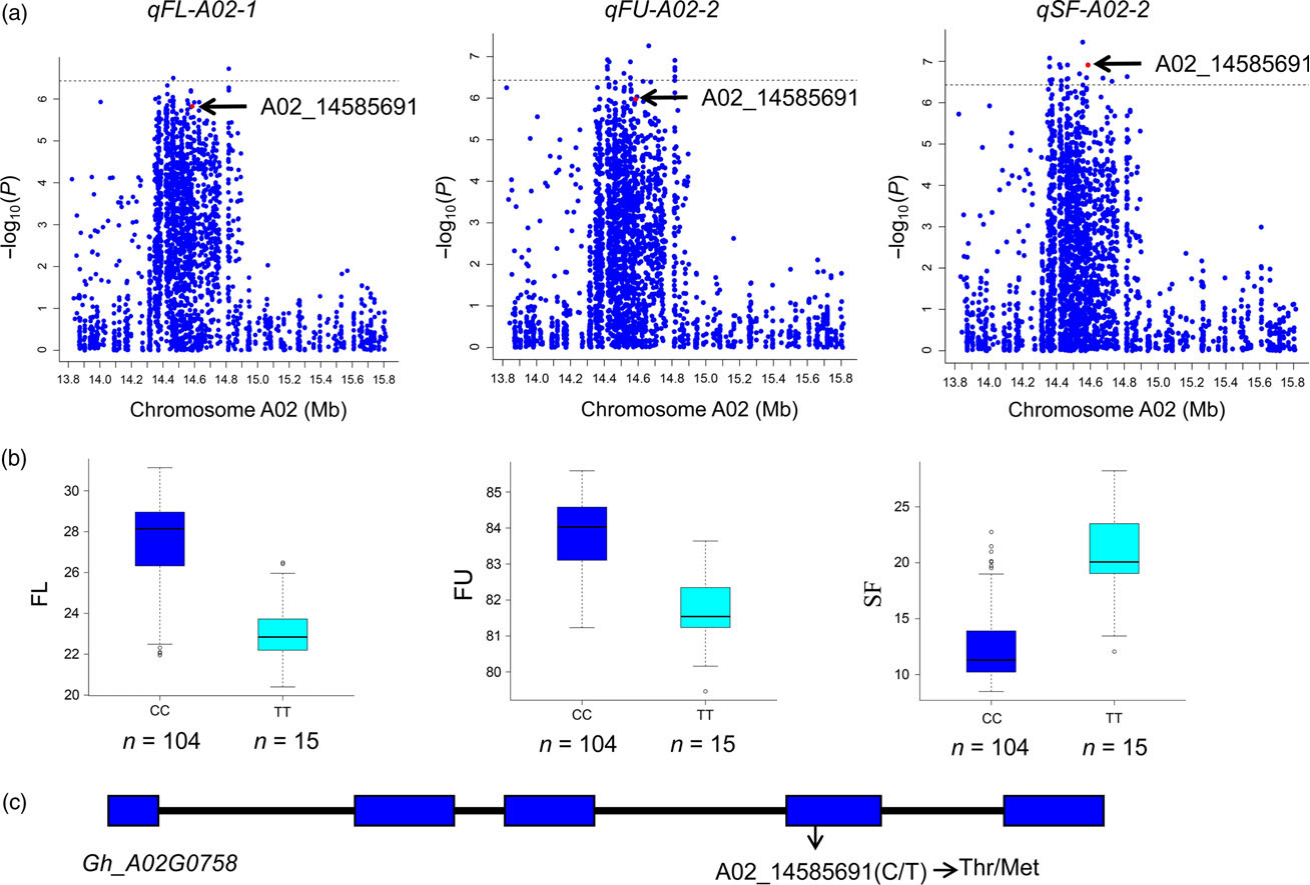
Identification of the *Gh_A02G0758* candidate gene. (a) QTLs of *qFL‐A02‐1*,*qFU‐A02‐2* and *qSF‐A02‐2* on chromosome A02 from 13.8 Mb to 15.8 Mb. Red dots indicate the SNP of A02_14585691. (b) Boxplots between the SNP of A02_14585691 and the fibre length (FL), fibre unity (FU) and short fibre (SF) traits. (c) SNP of A02_14585691 in the *Gh_A02G0758* gene. Thr, threonine; Met, methionine.

Most of the significant SNPs associated with QTLs were located in intergenic regions; however, some were located in the body of a gene, even causing amino acid mutations. For example, the SNP D11_58410018, which is related to the micronaire value (MV), on chromosome D11 is a splice site mutation resulting in a methionine‐to‐valine substitution (Table S17). For the SCW and FE traits, no QTLs were detected, but eight QTLs for LP and seven QTLs for SF were identified, suggesting that SF and LP are complex traits affected by multiple QTLs. Three QTLs, *qFU‐A02‐2*,* qSF‐A02‐2* and *qFL‐A02‐1,* were located in A02: 14.2–14.9 Mb and were significantly associated with the FU, SF and FL, respectively (Figure 6a,b). In this region, the SNP of A02_14585691 in *Gh_A02G0758* causes a mis‐sense variant, inducing an amino acid change from threonine to methionine (Figure 6c); the favourable allele was A02_14585691‐CC, whereas A02_14585691‐TT was missed from most of the accessions. The protein of *Gh_A02G0758* is an EamA‐like transporter family in *Arabidopsis thaliana* and serves as a UDP‐galactose transporter in cell wall synthesis (Zhang *et al*., 2015).

### Pleiotropy of QTLs

**Figure 7:**
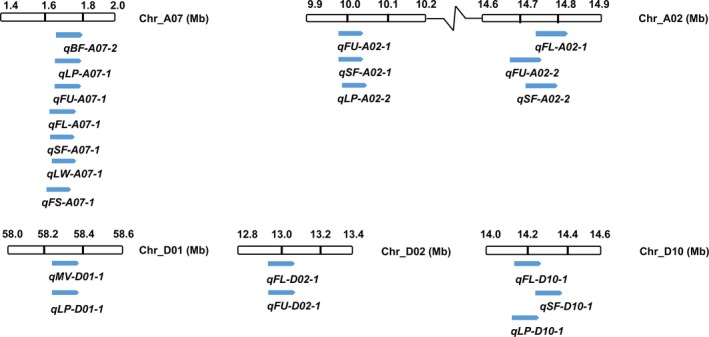
QTLs identified through association analysis overlap on the respective chromosomes.

Anchoring the QTLs on the chromosomes revealed that six regions contained more than one QTL each, which might be due to linkage or pleiotropy (Figure 7). Chromosome A07: 1.6–1.86 Mb harboured *qBF‐A07‐2*,* qLP‐A07‐1*,* qFU‐A07‐1*,* qFL‐A07‐1*,* qSF‐A07‐1, qLW‐A07‐1* and *qFS‐A07‐1*. The most significant SNPs among these seven QTLs were all located on SCAFFOLD1921_A07: 46 075–89 882 (<50 kb), which indicates that *qBF‐A07‐2* is pleiotropic and affects other fibre traits. The SI was negatively correlated with the FL, FU, FS, SCW, LP and LW, but positively correlated with SF and FE, which indicated that the brown fibre colour facilitated the production of SF and promoted the FE, but decreased the number, length and strength of the long fibre (Figure S26).

Additionally, chromosome A02: 10 002 642–10 046 008 contained *qFU‐A02‐1, qSF‐A02‐1* and *qLP‐A02‐2*; three QTLs overlapped around A02: 14.75 Mb; *qMV‐D01‐1* and *qLP‐D01‐1* were located at the same SNP of D01_58358869; *qFL‐D02‐1* and *qFU‐D02‐1* were located in the intergenic region between *Gh_D02G0795* and *Gh_D02G0796*; three QTLs, *qFL‐D10‐1*,* qSF‐D10‐1* and *qLP‐D10‐1*, were located in D10: 14.1–14.4 Mb (Figure 7). It is common for regions with overlapping QTLs on a chromosome resulting from linked or pleiotropic genes, particularly at the *qBF‐A07‐2* locus.

### Genetic basis of brown fibre cultivars

To identify the inheritance of favourable QTLs in two dark brown and 16 light brown cultivars, we checked 29 QTLs for fibre traits and two QTLs for brown fibre in them (Table S18). Z98 (Zhongmian 81) belongs to the dark brown cultivar. This cultivar possessed the favourable allele of these QTLs, but the QTLs overlapped with colour‐increasing allele of *qBF‐A07‐2*, which accelerates the accumulation of fibre pigment while exerting negative effects on other traits (Table S18). Thus, the fibre quality and yield of this cultivar was worse than that of the light brown cultivars. However, another dark brown cultivar, Z91 (Xincaimian 5), did not habour the colour‐increasing allele of *qBF‐A07‐2*, and some QTLs for fibre traits were different from Z98, which may be resulted from that it shares the same brown parent (Zong9802) with two light brown cultivars (Z95 and Z96).

The light brown fibre cultivars possessed most of the favourable QTLs for fibre traits and the colour‐decreasing allele of *qBF‐A07‐2*. The fibre quality and yield of these light brown cultivars was better than the dark brown cultivars and similar to the white fibre group; even some of them were better than the white fibre group for the fibre traits (Table S18). However, Z68 retained the negative allele of *qLW‐A02‐1*, indicating that there is a potential ability to improve the brown fibre yield. Among the cultivars, Z61 (Xincaimian 28), a newly authorized cultivar in Xinjiang in 2017, aggregated all the favourable alleles of these QTLs so that it showed a better fibre quality and yield than the white fibre group in most fibre traits.

## Discussion

### Abnormal recombination of the *Lc*_*1*_ region

In the linkage map of the *Lc*_*1*_ region (Figure 1c), a long cosegregating region was identified on chromosome A07, which reduced the fine‐mapping resolution of the target gene. A genomic inversion event around *Lc*_*1*_ has been reported previously (Hinchliffe *et al*., 2016), which might cause the lower recombination rate. Such a long cosegregating region could easily cause linkage drag, which is disadvantageous in the modification of a target trait because of the unfavourable QTLs linked with the favourable gene (Voss‐Fels *et al*., 2017). This finding will allow us to avoid genetic drag in the genetic improvement of brown fibre. Furthermore, a recombination hotspot was located at the right boundary of *Lc*_*1*_. Recombination hotspots are found and distributed at random across the genomes of different species (Drouaud *et al*., 2006; Dumont *et al*., 2011; Myers *et al*., 2005; Paul *et al*., 2016). Recombination might induce changes in agronomic traits and genomic diversity (Pan *et al*., 2016). Brown fibre is common in various cotton species (Carvalho *et al*., 2014; Hutchinson, 1946; Murthy, 2001; Ware, 1932), and this recombination hotspot might induce the production of brown fibre via hybridization between different cotton lines. The high polymorphisms of the *Gh_A07G2341* candidate gene for *qBF‐A07‐1* might be induced by this recombination hotspot (Figure 3e and Table S8). In general, we hypothesized that the recombination hotspot induced genetic variation and genomic inversion and the inversion gave rise to the cosegregating region.

### Population structure of brown fibre cotton

A population structure mostly results from geographic isolation and gene exchange isolation, and it is an important factor in genome‐wide association analysis (Huang *et al*., 2017). In this study, a distinctive population structure was found between the brown and white fibre accessions (Figure 2); furthermore, a genome‐wide analysis of selection signatures showed that many SNPs (*F*_ST_ > 0.5) were significantly differentiated between the brown and white fibre cotton groups (Tables S14 and S15). The haploid analysis of significant association signals of the *qBF‐A07‐1* and *qBF‐A07‐2* loci (Tables S6,S7,S11,S12) indicated that most of the brown cotton accessions have retained the introgression signature of *G. barbadense*, suggesting that the large set of differentiated SNPs across the genome might derive from a distant hybridization between *G. hirsutum* and *G. barbadense*, causing the distinctive population structure observed between the white and brown fibre cotton accessions. To exclude the effect of population structure in the association analysis, the MLM (P+Q+K) was selected to conduct association mapping the fibre traits in multiple environments (Bradbury *et al*., 2007). The results suggest that the MLM (P+Q+K) can effectively avoid false positives and uncover the genetic basis of complex traits (Figures S12–S25).

### Genetic dissection of *qBF‐A07‐1* and *qBF‐A07‐2* from the *Lc*_*1*_ region

In most studies, natural brown cotton has been treated as a qualitative trait, but the colour of natural brown cotton ranges from light brown to dark brown. To genetically understand this variation, brown cotton was evaluated as a qualitative trait in the present study through case–control association (Figure 3). Furthermore, the SI of brown fibre was quantified (Figure 4a). Finally, two loci, *qBF‐A07‐1* and *qBF‐A07‐2*, were identified from the *Lc*_*1*_ region. The QTL of *qBF‐A07‐1* mediates the production of brown fibre, and the QTL of *qBF‐A07‐2* induces the variation of brown colour. These two loci act as an integrated locus in the linkage population due to a long cosegregating region. During the domestication and breeding of brown fibre cotton, these two loci might have been gradually separated in the natural brown fibre population. To validate these two loci, a genome‐wide selection signature analysis was performed to verify the existence of these two loci in the *Lc*_*1*_ region (Figure 5).

The QTL of *qBF‐A07‐1* was fine‐mapped to *Gh_A07G2341*, which encodes an R2R3‐MYB transcription factor that was previously reported to be up‐regulated in brown fibre (Hinchliffe *et al*., 2016). MYB transcription factors are the main regulatory factors in the flavonoid pathway, and they can combine proteins of the WD40 and bHLH families to regulate expression levels in the flavonoid pathway (Koes *et al*., 2005; Xu *et al*., 2015). A number of studies have shown that the transcripts of the structural genes *C4H*,* CHS*,* F3′H* and *F3′5′H* are highly expressed in the brown fibre of cotton (Feng *et al*., 2013; Hinchliffe *et al*., 2016; Tan *et al*., 2013). Abundant polymorphisms have been identified in *Gh_A07G2341*; in the future, we can conduct experiments to locate the natural functional variation in this gene. Interestingly, the significantly associated SNP of A07_1227674 in *qBF‐A07‐2* caused a mis‐sense variant; this SNP belonged to *Gh_A07G0100,* which encodes a WD40 protein. The MYB‐bHLH‐WDR protein complex has been reported to regulate the flavonoid pathway (Xu *et al*., 2015), suggesting that these two candidate genes might form a protein complex to affect the flavonoid pathway.

### Relationship between agronomic traits and the SI of brown fibre

Compared with other association analysis of fibre traits (Huang *et al*., 2017; Wang *et al*., 2017), some new QTLs related to fibre quality and yield were discovered in this study, and favourable alleles have been enriched in the elite cultivars (Table S18). Due to the pleiotropy of QTLs, some QTLs overlapped within a given region of a chromosome, such as *qFU‐A02‐2*,* qSF‐A02‐2* and *qFL‐A02‐1*; and the *Gh_A02G0758* gene has been identified as the candidate gene for these three QTLs (Figure 6). The pleiotropy of QTLs is beneficial for the confirmation of functional loci associated with different traits, and this finding is particularly obvious in the region of *qBF‐A07‐2*, which harbours a QTL that significantly affects the colour of brown fibre and overlaps with *qLP‐A07‐1*,* qFU‐A07‐1*,* qFL‐A07‐1*,* qSF‐A07‐1, qLW‐A07‐1* and *qFS‐A07‐1* (Figure 7). This finding indicates that *qBF‐A07‐2* causes variation in brown fibre, while exerting a negative effect on fibre traits. Therefore, focusing on this QTL to modify the brown fibre will be useful in future work.

## Conclusion

Based on linkage and association mapping, the *Lc*_*1*_ region has been dissected into two loci contributing to brown fibre, one is responsible for colour generation and the other is responsible for colour variation. Most tetraploid modern brown cotton cultivars have the introgression signature of *G. barbadense*. QTL mapping revealed that the QTL responsible for colour variation is pleiotropic to many fibre‐related QTLs and negatively affects fibre traits, suggesting that we must balance colour with fibre quality and yield to breed elite brown fibre cultivars similar to white fibre cotton. This study provides a clear description of the genetics of brown fibre cotton.

## Experimental procedures

### Plant materials

To fine‐map the brown fibre locus, *G. hirsutum* acc. Handan208 and *Youse* (*ys*) (Figure S1), a dark brown fibre mutant resulting from distant hybridization between *G. barbadense* acc. Pima90‐53 and Handan208, were crossed to generate three F_2_ populations. A total of 3990 F_2_ plants, including 1019 recessive plants (white fibre plants), were finally used to fine‐map the brown fibre locus. From the three F_2_ linkage populations, 243 individuals were planted in Wuhan, Hubei Province, China (2015); 1578 individuals were planted in Sanya, Hainan Province, China (2016); and 2169 individuals were planted in Wuhan, Hubei Province, China (2016). To test the segregation ratio of the brown fibre locus, the colour of the fibre was distinguished as white, light brown and dark brown in the segregating populations (Table S1). To test the expression of the candidate genes in brown fibre, cotton fibres were collected from HD208, Pima90‐53 and *ys* at 5 days post anthesis (DPA).

To generally study brown fibre cotton, 100 brown fibre accessions (*G. hirsutum*) were collected from the Institute of Cotton Research, Shihezi Academy of Agricultural Sciences, Xinjiang. The pedigree of 51 accessions introduced from breeding institutes in China was unknown due to the old age of breeding of these accessions. Additionally, 109 white fibre cotton (*G. hirsutum*) accessions that were previously used in association mapping in our laboratory were selected for the population structure and selection signature analyses (Huang *et al*., 2017; Nie *et al*., 2016; Wang *et al*., 2017). The 100 brown fibre accessions and 21 white fibre accessions selected from the 109 white fibre accessions based on different cultivation areas and genetic diversity of SSR markers (Nie *et al*., 2016) were phenotyped for the colour, yield and fibre quality traits (Tables S3 and S4).

### Phenotyping and statistics of the phenotypic data

Phenotyping was conducted with the 121 accessions and collected from multiple environments and field experiment locations, including Huanggang, Hubei Province, China in 2015; Shihezi, Xinjiang Province, China in 2015 and 2016; and Ezhou, Hubei Province, China (destroyed by flooding and waterlogging). In each environment, the 121 accessions were planted with two replicates; in each replicate, these accessions with ten plants in one row were randomly planted. Twenty bolls were collected from the middle fruit branches of each row for trait analysis. Before ginning, the SCW, one of the yield traits, was evaluated. After ginning, two additional yield traits, the LW and the LP, were evaluated. To test the fibre quality, 10–15 g of fibre from each sample was sent to the Institute of Cotton Research, Shihezi Academy of Agricultural Sciences, Xinjiang. The fibre quality traits including FL, FE, MV, FU, SF and FS were tested at 20 °C under 65% relative humidity with an HVI1000 Automatic Fibre Determination System (User technologies, Inc., USTER, Switzerland).

To evaluate the SI of the 121 accessions, the seed fibres of three cotton bolls collected from each accession were cleaned with a comb, pasted onto white paper using glue and scanned in a scanner with a standard model (Figure S2). Adobe Photoshop (version 2.0) was applied to measure the fibre colour in the scanned images via the CIE (International Commission on Illumination, Vienna, Austria) L*A*B model (Ibraheem *et al*., 2012). In the CIE L*A*B colour space, L indicates whiteness, A indicates the colour from green to red, and B indicates the colour from blue to yellow. Moreover, the SI of 121 accessions was measured using the following formula: SI = [(ΔL)^2 + (ΔA)^2 + (ΔB)^2]^(1/2) (Δ is the difference between the sample and control) (Melgosa *et al*., 1996), with the colour space of HD208 set as the control value. An ANOVA of SI across multiple environments was performed with the R function.

The phenotypic data from multiple locations and years were fitted using an R script applying the BLUP method (Huang *et al*., 2017). The mean values of two replicates from the same location and year were calculated using Microsoft Excel for application in a one‐year/one‐location genome‐wide analysis.

### Linkage mapping and expression analysis

For the linkage population, sample DNA was extracted using the CTAB method and genotyped using a ZAG (Zero Agarose Gel)™ system. A total of 2000 SSR markers from the genetic map constructed in our laboratory were used to screen markers between parents (Li *et al*., 2016b). Polymorphic markers between parents were screened for linkage markers of the brown fibre gene according to the bulked segregation method (BSA) (Michelmore *et al*., 1991). DNA from 20 individuals with dark brown fibre was mixed to build the brown bulk, and DNA from 20 individuals with white fibre was mixed to build the white bulk. The recessive plants from the F_2_ population were genotyped using the polymorphic markers between the two bulks (Zhang *et al*., 1994).

In addition to the above markers, Primer3 software (Koressaar and Remm, 2007) and the published cotton genome sequence (*G. hirsutum*) (Zhang *et al*., 2015) were applied to develop UTR and SSR markers (Table S19). These markers were applied to genotype the recessive F_2_ plants. A final genetic map of the brown fibre gene was constructed using Mapmaker 3.0 (Lander *et al*., 1987) and MapChart (Voorrips, 2002) according to the genotypes.

The recombination rate was calculated using the function Re = genetic distance/physical distance (Myers *et al*., 2005), and the average recombination rate of chromosome A07 was calculated by applying the published genetic map of chromosome A07 in cotton (Li *et al*., 2016b). Recombination hotspot regions were defined based on a recombination rate that was 10‐fold higher than the average recombination rate.

To confirm the expression level of the candidate genes, the total RNA from fibres at five DPA was extracted using the DP432 plant RNA kit (Tiangen Biotech, Beijing). Approximately 3 μg of total RNA was reverse‐transcribed using SuperScript III reverse transcriptase (Invitrogen, Cat. No. 18080‐093, Waltham, MA) in a 20‐μL reaction mixture to obtain cDNA. The primers employed for reverse transcription PCR (RT‐PCR) are shown in Table S19.

### Genotyping the association panel via resequencing

The brown fibre accessions were planted in a field in Wuhan, Hubei Province, China (2016), and total plant genomic DNA was extracted from each accession. The DNA quality was checked in an agarose gel, and the concentration was determined using a NanoDrop 2000. DNA from each accession was employed to construct a library and was sequenced to a sixfold depth using a HiSeq 2000 instrument. The sequence data for the 100 brown cotton accessions are available in the NCBI Sequence Read Archive (SRA) under accession number PRJNA412456 (Table S20). The 109 accessions of white fibre cotton were resequenced in a previous project (Table S20) (Wang *et al*., 2017). Clean reads were obtained by filtering the raw sequence data of each accession. Paired‐end sequence reads were aligned against the reference genome sequence (*G. hirsutum* acc. TM‐1) (Zhang *et al*., 2015) employing BWA software. SNP calling was conducted using the Genome Analysis Toolkit (version 3.1.1) and SAMtools/BCFtools software (Li, 2011; Li *et al*., 2009), using the specific steps and parameters described previously (Shen *et al*., 2017).

### Population structure, case–control association and selection signature analysis of the 209‐accession panel

To evaluate the population structure of the 100 brown fibre and 109 white fibre accessions, the Structure 2.3.4 software was utilized (Evanno *et al*., 2005) with 50,000 homozygous SNPs to compute the correct number of subgroups (*K* value). The *K* value was set from 1 to 10, with five independent repeats. The natural logarithms of the probability data (LnP(*K*)) and the ad hoc delta *K* statistical were calculated using STRUCTURE HARVESTER (Earl and Vonholdt, 2011), and the optimal *K* according to the delta *K* value was then selected (Mezmouk *et al*., 2011). Finally, the Q matrix was obtained by integrating five independent replicate runs and applying CLUMPP software (Jakobsson and Rosenberg, 2007).

Principal component analysis (PCA) and a phylogenetic tree of the 209‐accession panel were computed using TASSEL (Bradbury *et al*., 2007) (version 5.0) with 50 000 homozygous SNPs. The phylogenetic matrix was obtained from the cladogram module of TASSEL by applying 50 000 homozygous SNPs. R software was used to draw the figure corresponding to the PCA plot and the phylogenetic tree.

For the 209‐accession panel, a total of 7 472 949 SNPs, with a minor‐allele frequency (MAF) > 0.05, were called. To control the quality of the SNPs and perform the case–control association analysis, the population structure of the 209 accessions obtained from the population structure calculation process was employed as the covariate to stratify the population, and Plink (Purcell *et al*., 2007) (version 1.07) was applied according to the instructions for the Plink pipeline.

All the homozygous SNPs from the 209 accessions were selected to identify a whole‐genome selection signature. The computation and production of the corresponding figures were realized within the R environment using the calculation steps described previously (Gondro *et al*., 2013).

### LD and GWAS of the 121‐accession panel

To calculate the LD across different chromosomes, the SNP density across chromosomes at the average level of 100 SNPs/Mb was retained. TASSEL (Bradbury *et al*., 2007) (version 5.0) was applied to calculate the *r* ^*2*^ parameter between pairs of SNPs. The *r* ^*2*^ value between pairs of SNPs was employed to calculate the LD of a chromosome (Vos *et al*., 2017).

An MLM (P+K+Q) was used to conduct an association analysis between the SNPs and phenotypes (P), including BLUP and three environmental traits. The pairwise relatedness coefficients (K, kinship matrix) were computed using TASSEL (version 5.0) and the population structure (Q matrix) obtained with Structure 2.3.4 software as described above. These two matrices were employed as fixed effects in the model to correct the stratification (Yu *et al*., 2006). The GWAS threshold of the MLM was set to −log_10_(1/*N*) (where N is the total number of SNPs used) according to the Bonferroni‐corrected thresholds, whereas the GLM was set to −log_10_(0.01/*N*) (Liu *et al*., 2015). The significant SNPs in multiple environments were integrated into QTLs according to an LD value on the chromosome. A conditional association analysis was applied to test the existence of two close QTLs. The concrete step was performed to set the top SNP of the QTL; this SNP was transformed into a population file format, and this file was then applied to stratify the association model. If other SNPs over the threshold, this region was assumed to exhibit two QTLs. Haplotype blocks were constructed using Haploview software with the confidence interval method (Barrett *et al*., 2005; Gabriel *et al*., 2002).

## Conflict of interest

The authors declare no conflict of interest.

## Supporting information


**Figure S1** Bolls of the *ys* mutant, F_1_ and HD208.
**Figure S2** Fibre colour of 100 brown fibre accessions.
**Figure S3** Haploid analysis of *Hap1* among *G. barbadense* acc. Pima90‐53, *G. hirsutum* cv. HD208 and the *ys* mutant.
**Figure S4** GWAS of the shade index (SI) in the BLUP data using the MLM (P+Q+K).
**Figure S5** GWAS of the shade index (SI) in the HG15 environment using the MLM (P+Q+K).
**Figure S6** GWAS of the shade index (SI) in the XJ15 environment using the MLM (P+Q+K).
**Figure S7** GWAS of the shade index (SI) in the BLUP data using the GLM.
**Figure S8** GWAS of the shade index (SI) in the HG15 environment using the GLM.
**Figure S9** GWAS of the shade index (SI) in the XJ16 environment using the GLM.
**Figure S10** Relationship between the significant SNPs and the shade index (SI).
**Figure S11** Boxplots of the nine agronomic traits among the dark brown fibre group (*n *=* *30), light brown fibre group (*n *=* *70) and white fibre group (*n *=* *109).
**Figure S12** GWAS of the fibre length (FL) in the BLUP data using the MLM (P+Q+K).
**Figure S13** GWAS of the fibre unity (FU) in the BLUP data using the MLM (P+Q+K).
**Figure S14** GWAS of the micronaire value (MV) in the BLUP data using the MLM (P+Q+K).
**Figure S15** GWAS of the short fibre percentage (SF) in the BLUP data using the MLM (P+Q+K).
**Figure S16** GWAS of the lint percentage (LP) in the BLUP data using the MLM (P+Q+K).
**Figure S17** GWAS of the fibre length (FL) in the HG15 environment using the MLM (P+Q+K).
**Figure S18** GWAS of the lint percentage (LP) in the HG15 environment using the MLM (P+Q+K).
**Figure S19** GWAS of the fibre unity (FU) in the XJ15 environment using the MLM (P+Q+K).
**Figure S20** GWAS of the micronaire value (MV) in the XJ15 environment using the MLM (P+Q+K).
**Figure S21** GWAS of the lint percentage (LP) in the XJ15 environment using the MLM (P+Q+K).
**Figure S22** GWAS of the lint weight (LW) in the XJ15 environment using the MLM (P+Q+K).
**Figure S23** GWAS of the fibre length (FL) in the XJ16 environment using the MLM (P+Q+K).
**Figure S24** GWAS of the fibre unity (FU) in the XJ16 environment using the MLM (P+Q+K).
**Figure S25** GWAS of the lint percentage (LP) in the XJ16 environment using the MLM (P+Q+K).
**Figure S26** Correlation matrix between the shade index (SI) and nine agronomic traits in 100 brown fibre accessions.

**Table S1** Chi‐square test in the F_2_ population across three years.
**Table S2** Calculation of recombination rates.
**Table S3** Information for the 100 brown fibre accessions (*G. hirsutum*).
**Table S4** Information for the 109 white fibre accessions (*G. hirsutum*).
**Table S5** PCA of 209 accessions.
**Table S6** Haploid analysis of *Hap1*.
**Table S7** Haploid analysis of *Hap2*.
**Table S8** Polymorphisms of *Gh_A07G2341* in the 209‐accession panel.
**Table S9** SNP density and LD across 26 chromosomes.
**Table S10** Analysis of variance (ANOVA) results of the shade index (SI).
**Table S11** Haploid analysis of *Hap3*.
**Table S12** Haploid analysis of *Hap4*.
**Table S13** Epistatic analysis between *qBF‐A07‐2* and *qBF‐A07‐1*.
**Table S14** SNPs with *F*_ST_ > 0.5 between dark brown and white fibre cotton.
**Table S15** SNPs with *F*_ST_ > 0.5 between light brown and white fibre cotton.
**Table S16** Statistics for the association panel with BLUP.
**Table S17** GWAS of the 121‐accession panel.
**Table S18** QTLs analysis of cultivars.
**Table S19** Primers employed in this research.
**Table S20** Summary of the NCBI accessions for all the re‐sequencing data in this study.
