# Supplementary material for: Linkage and association mapping reveals the genetic basis of brown fibre (Gossypium hirsutum)
Source: Plant Biotechnol J. 2018 Mar 24;16(9):1654–66. doi: 10.1111/pbi.12902 (PMC6097129; doi:10.1111/pbi.12902)
Supplement: Supplementary file 1 — Figure S1 Bolls of the ys mutant, F1 and HD208. Figure S2 Fibre colour of 100 brown fibre accessions. Figure S3 Haploid analysis of Hap1 among G. barbadense acc. Pima90‐53, G. hirsutum cv. HD208 and the ys mutant. Figure S4 GWAS of the shade index (SI) in the BLUP data using the MLM (P+Q+K). Figure S5 GWAS of the shade index (SI) in the HG15 environment using the MLM (P+Q+K). Figure S6 GWAS of the shade index (SI) in the XJ15 environment using the MLM (P+Q+K). Figure S7 GWAS of the shade index (SI) in the BLUP data using the GLM. Figure S8 GWAS of the shade index (SI) in the HG15 environment using the GLM. Figure S9 GWAS of the shade index (SI) in the XJ16 environment using the GLM. Figure S10 Relationship between the significant SNPs and the shade index (SI). Figure S11 Boxplots of the nine agronomic traits among the dark brown fibre group (n = 30), light brown fibre group (n = 70) and white fibre group (n = 109). Figure S12 GWAS of the fibre length (FL) in the BLUP data using the MLM (P+Q+K). Figure S13 GWAS of the fibre unity (FU) in the BLUP data using the MLM (P+Q+K). Figure S14 GWAS of the micronaire value (MV) in the BLUP data using the MLM (P+Q+K). Figure S15 GWAS of the short fibre percentage (SF) in the BLUP data using the MLM (P+Q+K). Figure S16 GWAS of the lint percentage (LP) in the BLUP data using the MLM (P+Q+K). Figure S17 GWAS of the fibre length (FL) in the HG15 environment using the MLM (P+Q+K). Figure S18 GWAS of the lint percentage (LP) in the HG15 environment using the MLM (P+Q+K). Figure S19 GWAS of the fibre unity (FU) in the XJ15 environment using the MLM (P+Q+K). Figure S20 GWAS of the micronaire value (MV) in the XJ15 environment using the MLM (P+Q+K). Figure S21 GWAS of the lint percentage (LP) in the XJ15 environment using the MLM (P+Q+K). Figure S22 GWAS of the lint weight (LW) in the XJ15 environment using the MLM (P+Q+K). Figure S23 GWAS of the fibre length (FL) in the XJ16 environment using the MLM (P+Q+K). Figure S24 GWAS of the fib [file PBI-16-1654-s002.pdf]

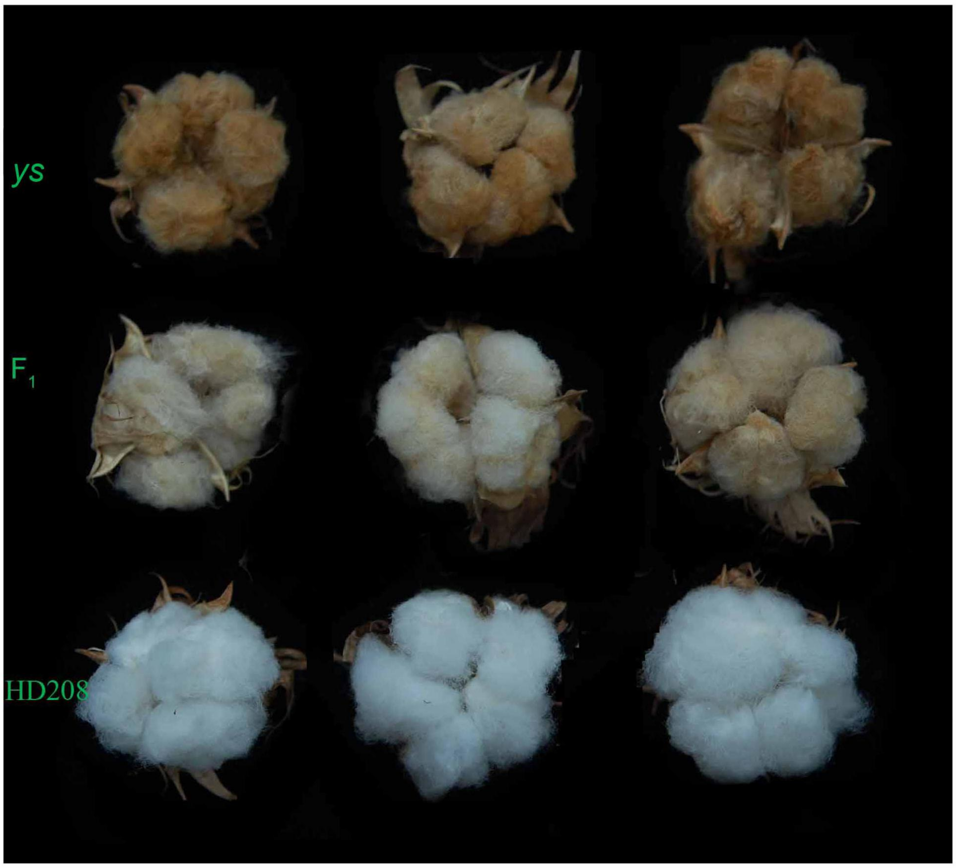

Figure S1. Bolls of the *ys* mutant, F_1 and HD208.

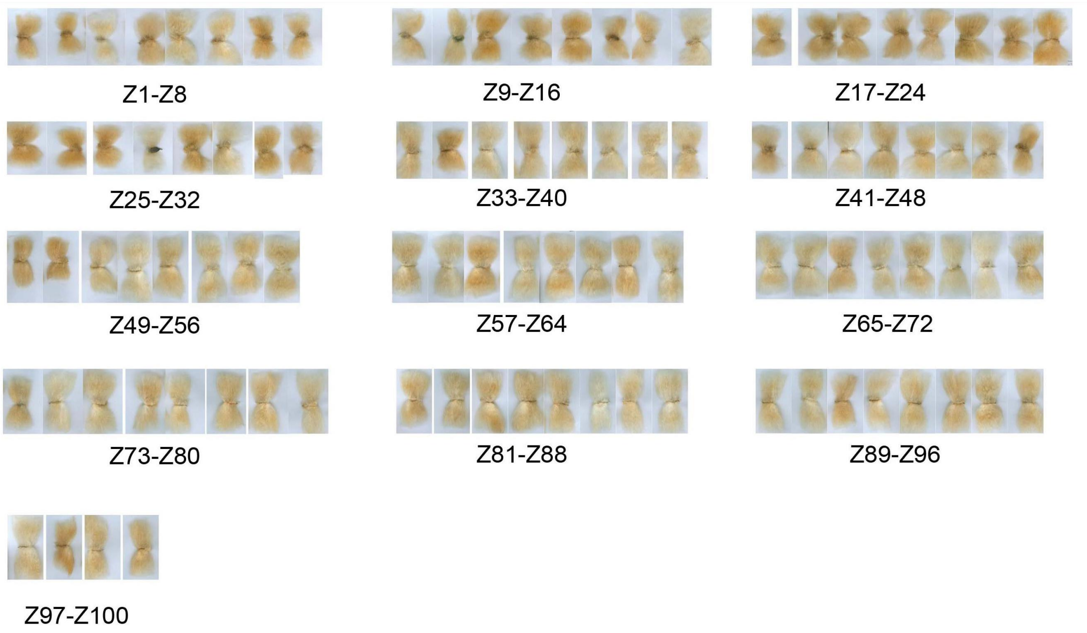

Figure S2. Fibre colour of 100 brown fibre accessions.

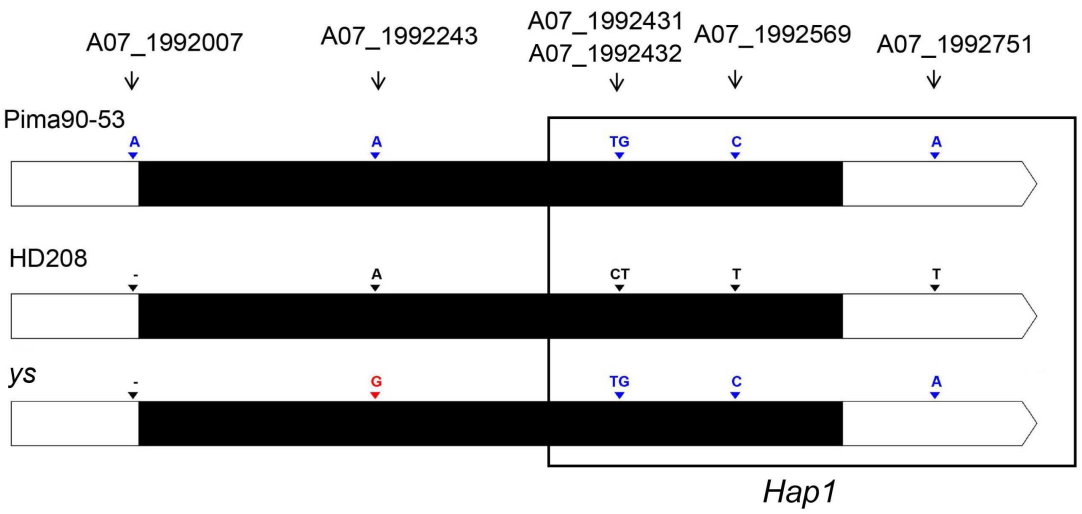

Figure S3. Haploid analysis of *Hap1* among *G. barbadense* acc. Pima90-53, *G. hirsutum* cv. HD208 and the *ys* mutant. SNPs in blue originate from Pima90-53; SNPs in black originate from HD208; SNPs in red are the new mutation; "-" in SNPs of A07_1992007 means the deletion of SNP. *Hap1* block has been indicated by box.

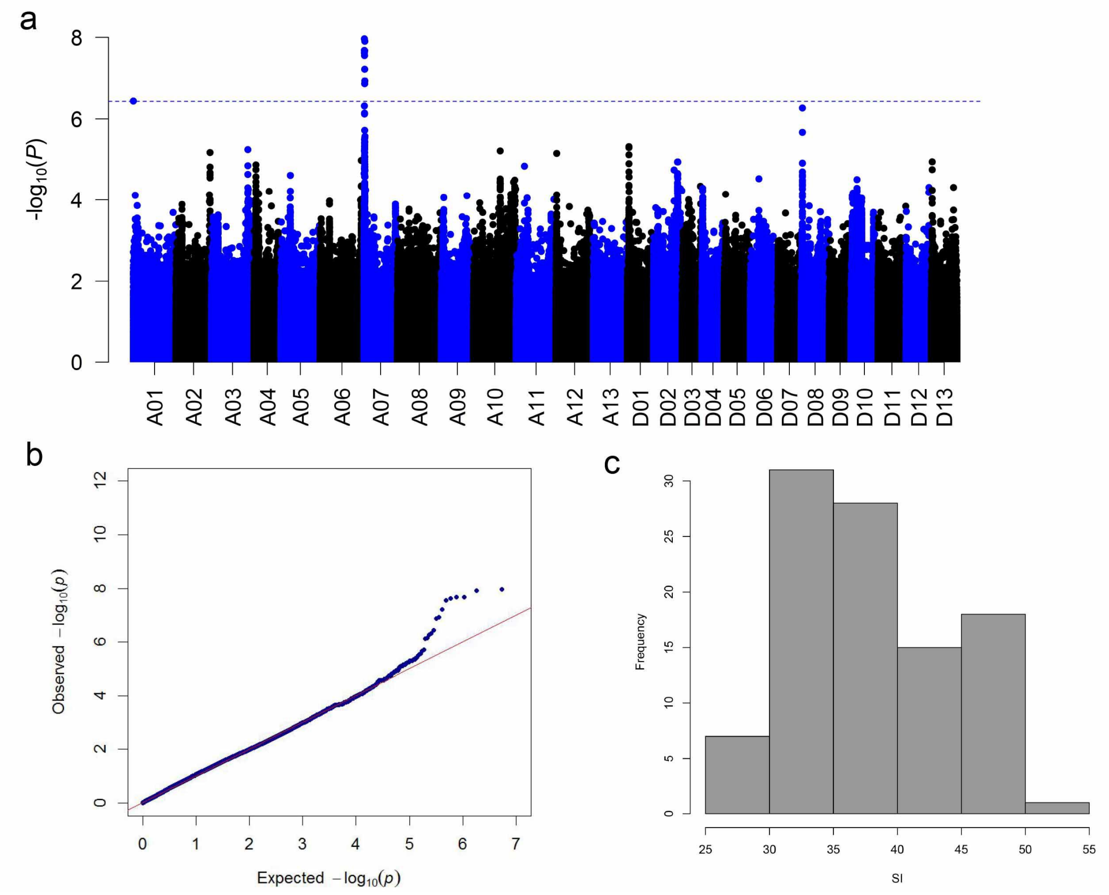

Figure S4. GWAS of the shade index (SI) in the BLUP data using the MLM (P+Q+K). (a) Manhattan plot of GWAS of SI. (b) Q-Q plot for SI. (c) Histogram of SI in the BLUP data.

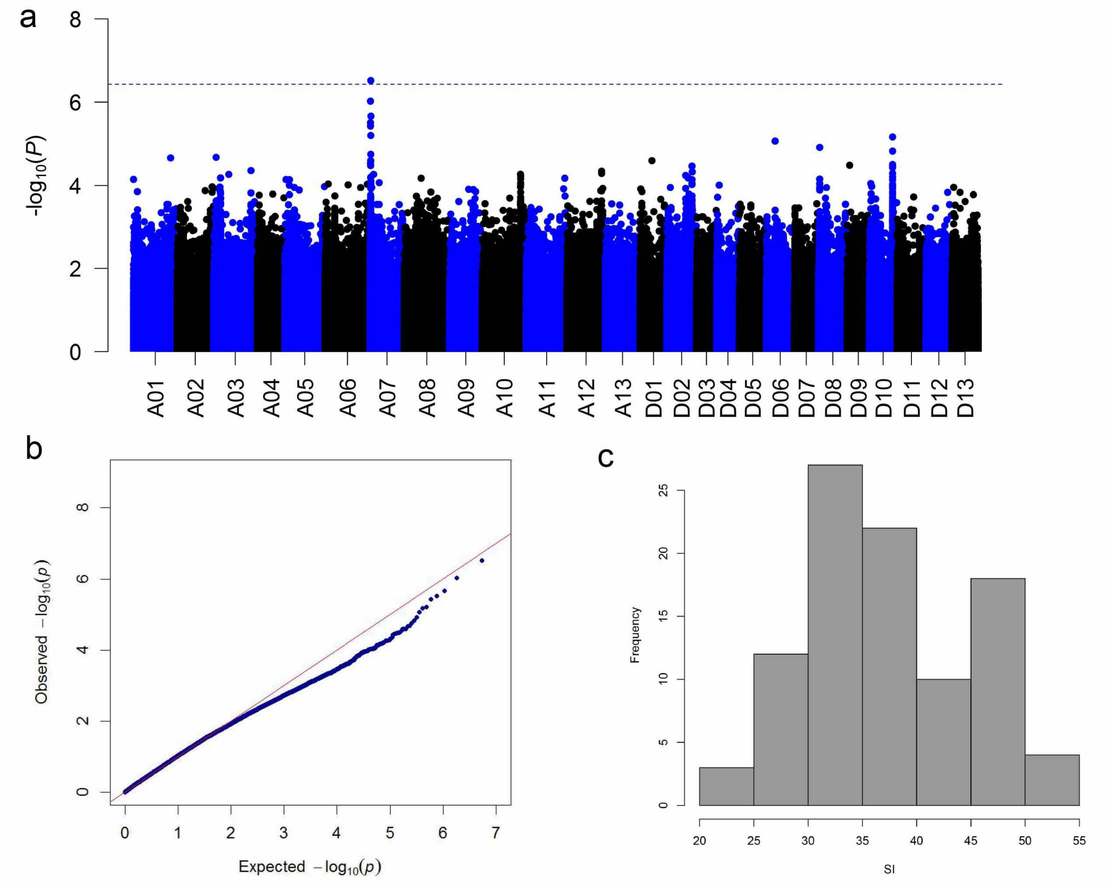

Figure S5. GWAS of the shade index (SI) in the HG15 environment using the MLM (P+Q+K). (a) Manhattan plot of GWAS of SI. (b) Q-Q plot for SI. (c) Histogram of SI in the HG15 environment.

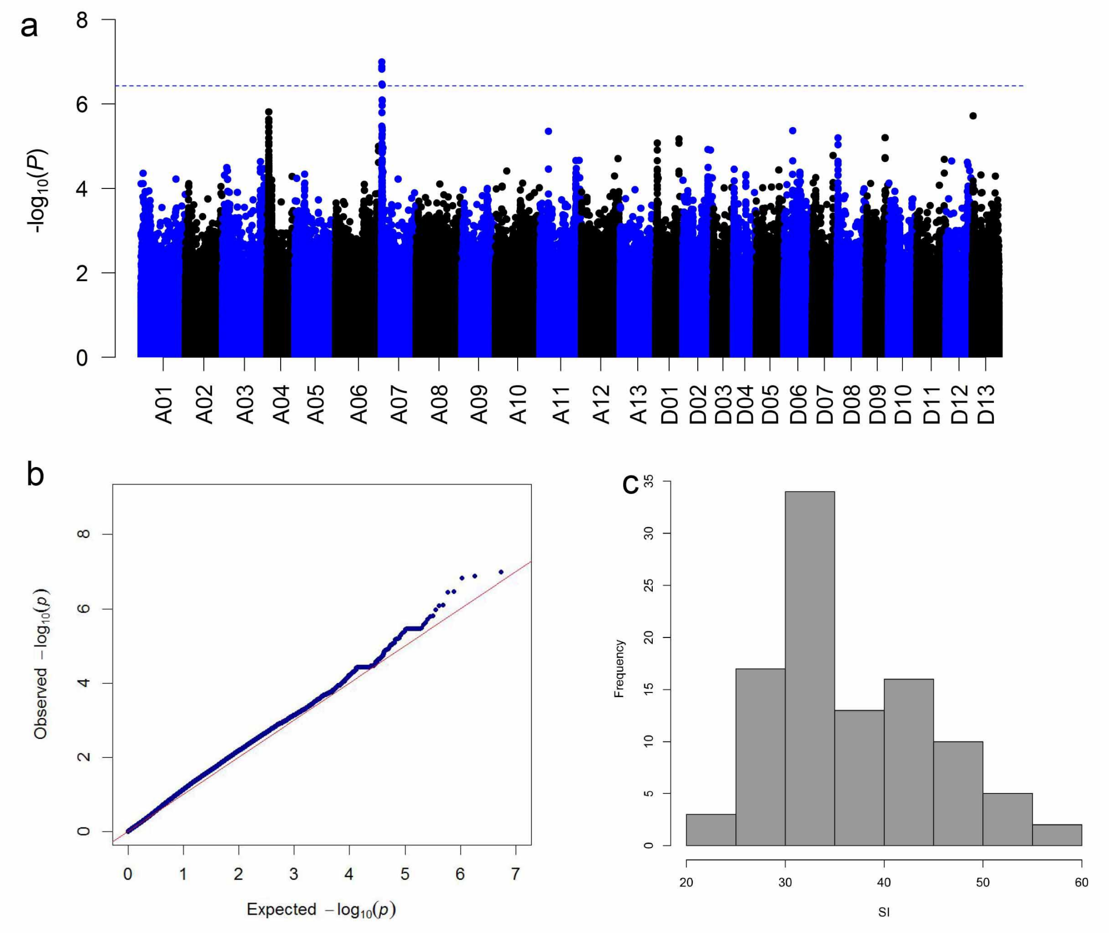

Figure S6. GWAS of the shade index (SI) in the XJ15 environment using the MLM (P+Q+K). (a) Manhattan plot of GWAS of SI. (b) Q-Q plot for SI. (c) Histogram of SI in the XJ15 environment.

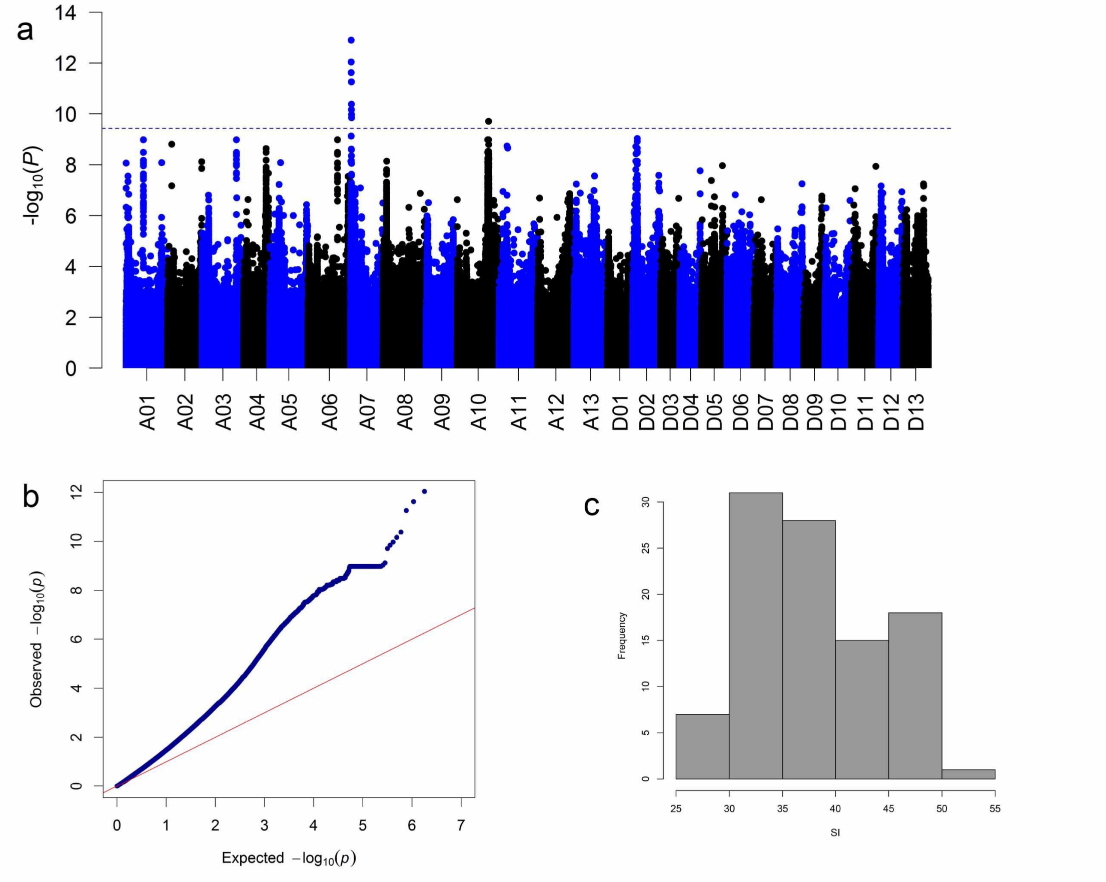

Figure S7. GWAS of the shade index (SI) in the BLUP data using the GLM. (a) Manhattan plot of GWAS of SI. (b) Q-Q plot for SI. (c) Histogram of SI of 100 brown fibre accessions in the BLUP data.

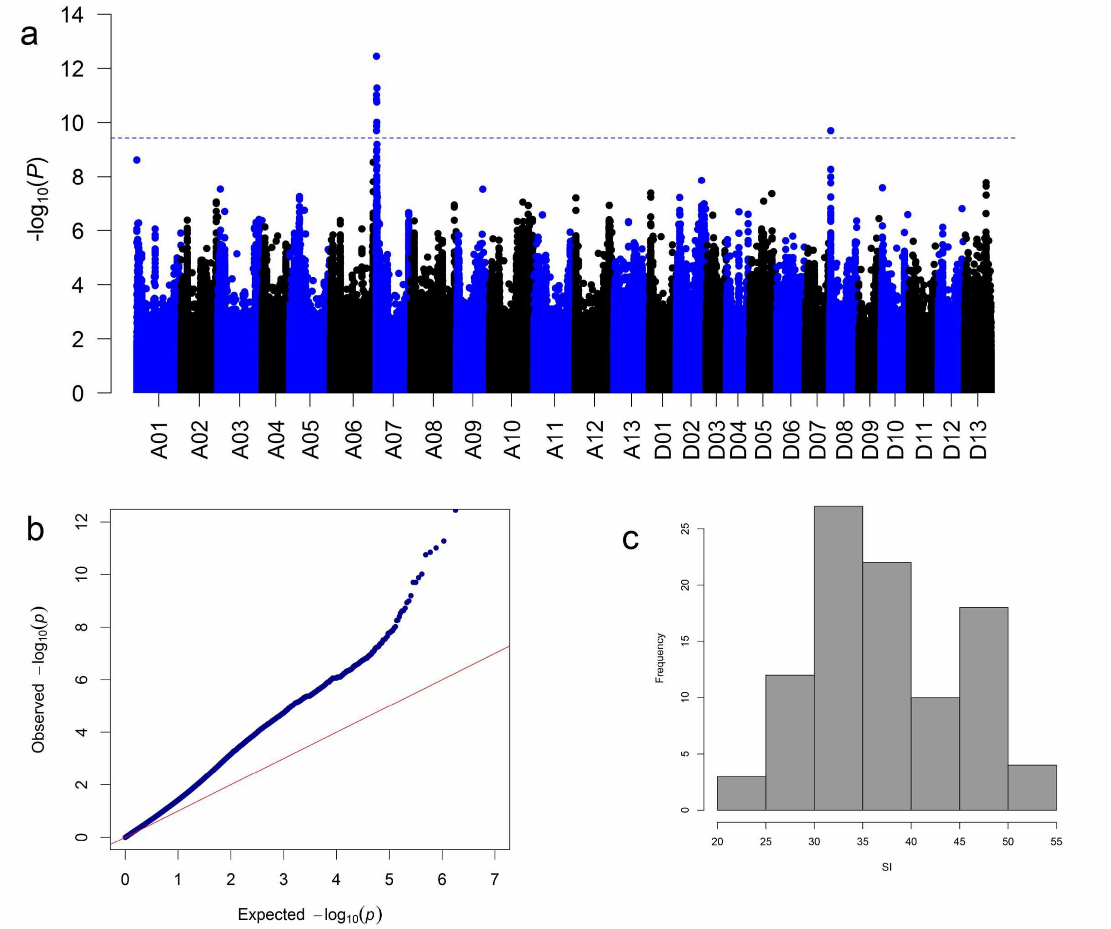

Figure S8. GWAS of the shade index (SI) in the HG15 environment using the GLM. (a) Manhattan plot of GWAS of SI. (b) Q-Q plot for SI (c) Histogram of SI of 100 brown fibre accessions in the HG15 environment using the GLM.

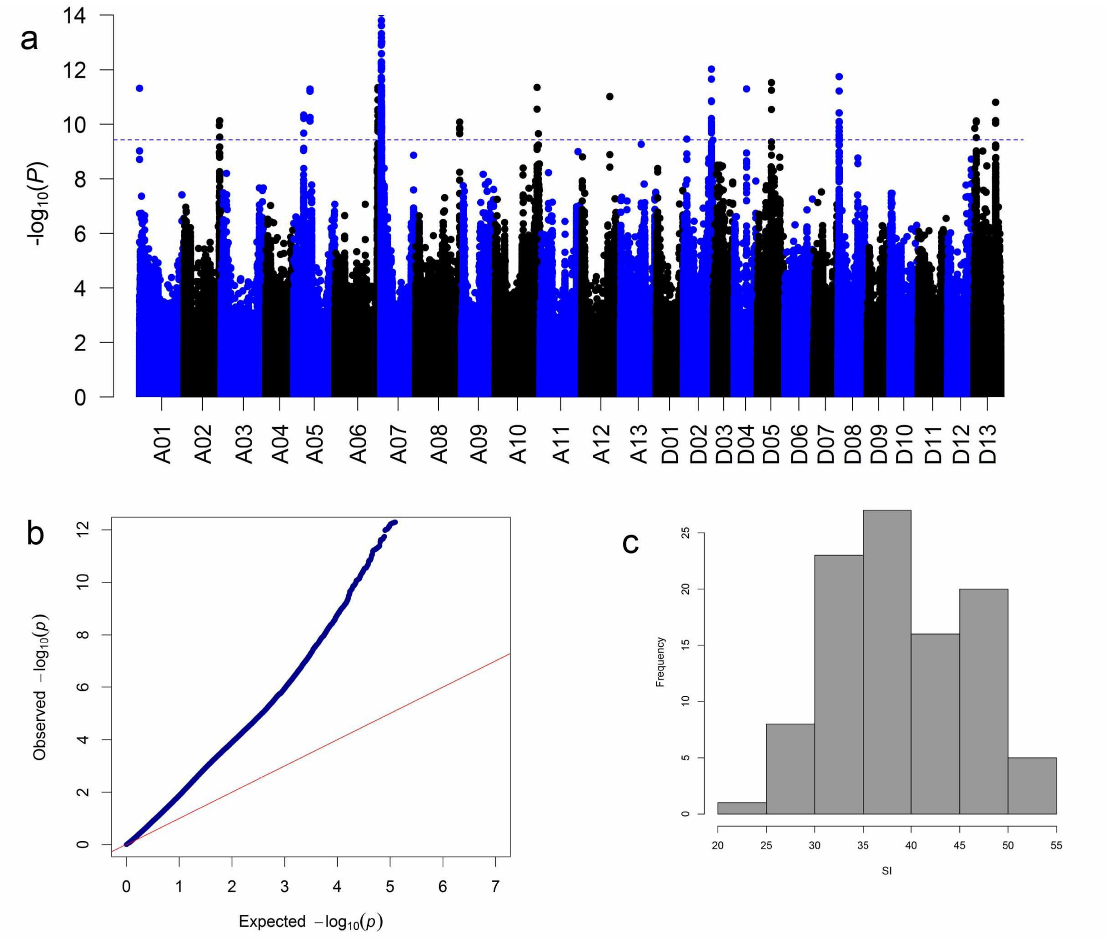

Figure S9. GWAS of the shade index (SI) in the XJ16 environment using the GLM. (a) Manhattan plot of GWAS of SI. (b) Q-Q plot for SI. (c) Histogram of SI of 100 brown fibre accessions in the XJ16 environment.

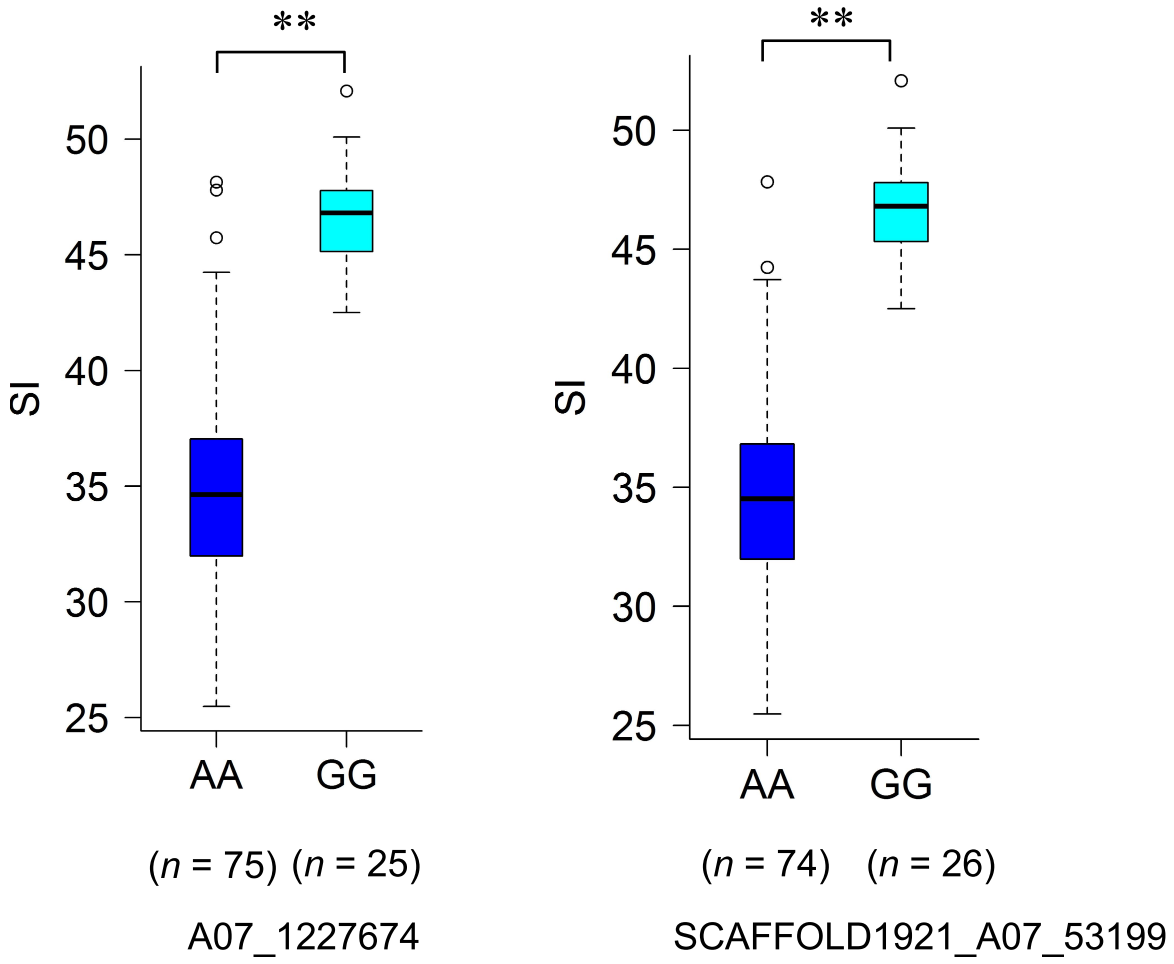

Figure S10. Relationship between the significant SNPs and the shade index (SI). (a) Boxplot between the SNP of A07_1227674 and SI. (b) Boxplot between the SNP of SCAFFOLD1921_A07_53199 and SI (***P* < 0.01, two tailed t-test).

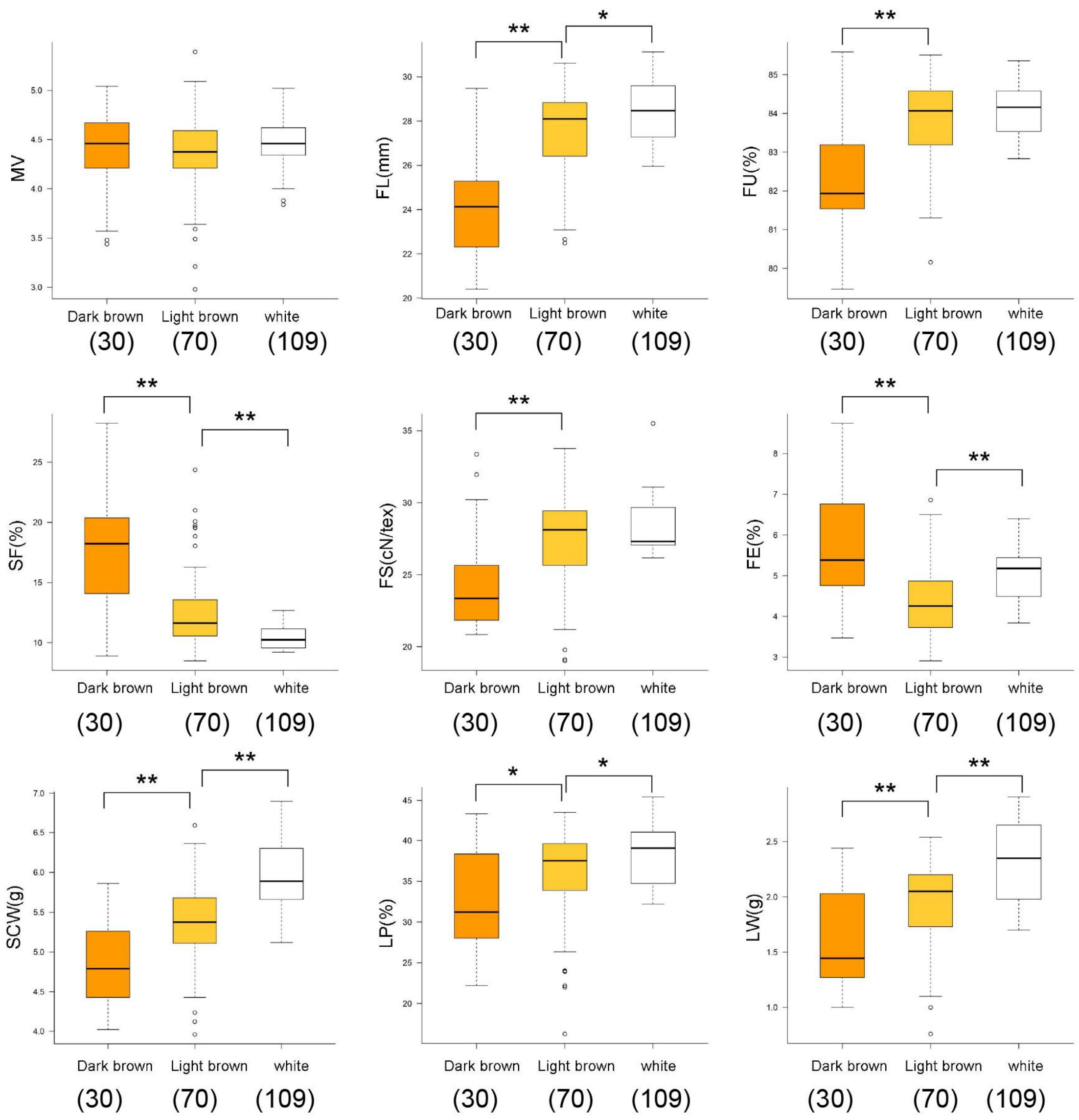

Figure S11. Boxplots of the nine agronomic traits among the dark brown fibre group (n = 30), light brown fibre group (n = 70) and white fibre group (n = 109), students t-test was performed to indicate the significant difference (*$p < 0.05$, **$p < 0.01$, two tailed t-test) between dark brown and light brown or between the light brown and white. Seed cotton weight, SCW; lint weight, LW; lint percentage, LP; fibre length, FL; fibre elongation, FE; micronaire value, MV; fibre uniformity, FU; short fibre, SF; fibre strength, FS.

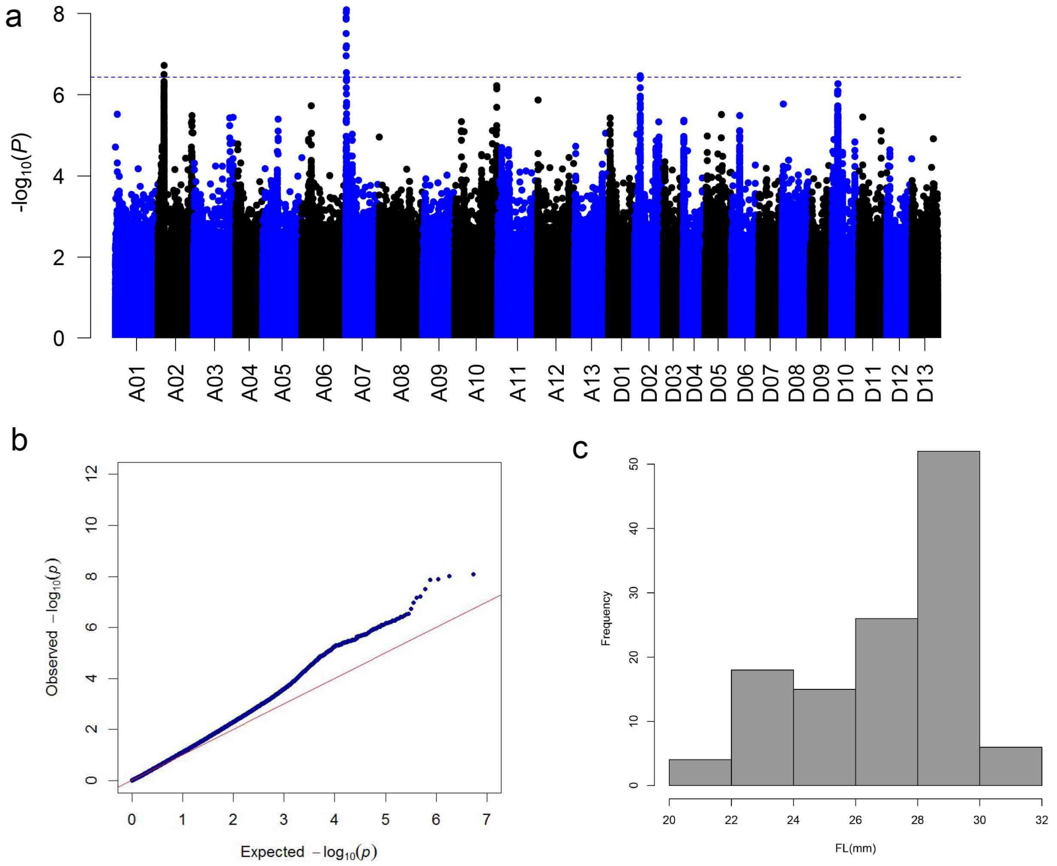

Figure S12. GWAS of the fibre length (FL) in the BLUP data using the MLM (P+Q+K). (a) Manhattan plot of GWAS of FL. (b) Q-Q plot for FL. (c) Histogram of FL in the BLUP data.

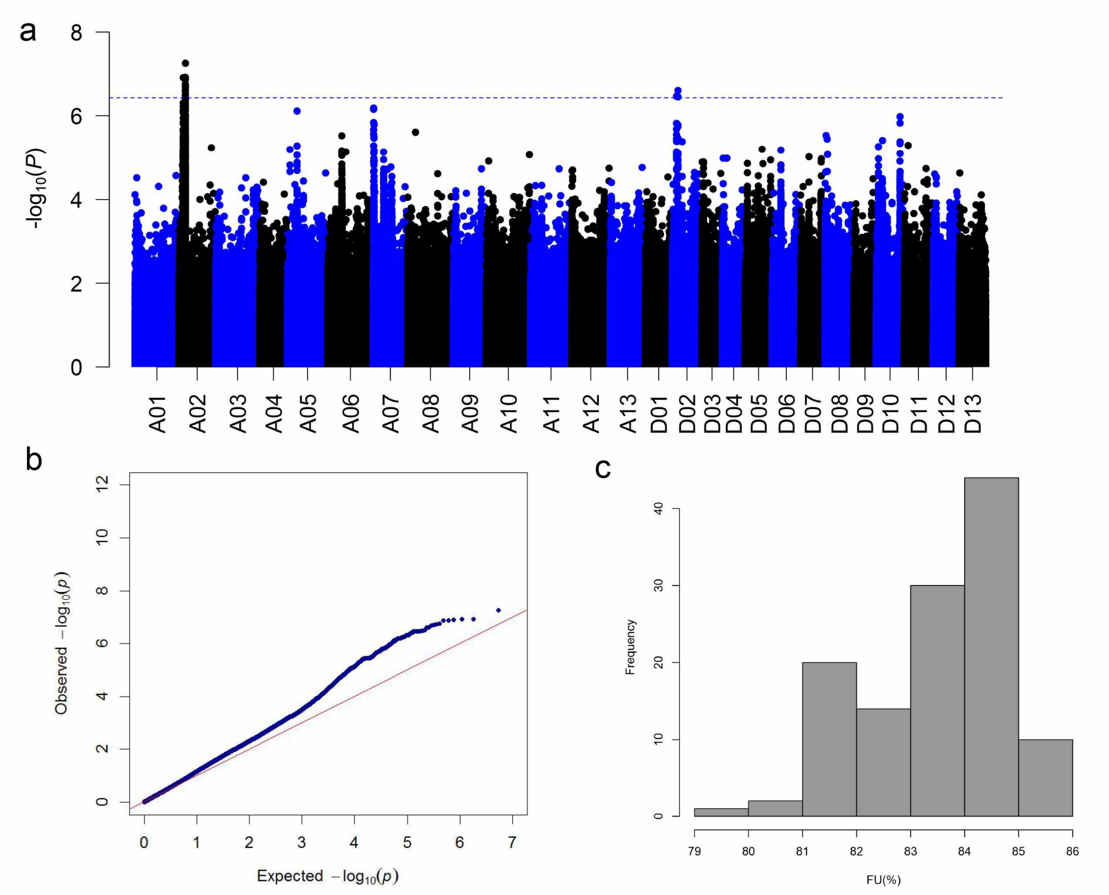

Figure S13. GWAS of the fibre unity (FU) in the BLUP data using the MLM (P+Q+K). (a) Manhattan plot of GWAS of FU. (b) Q-Q plot for FU. (c) Histogram of FU in the BLUP data.

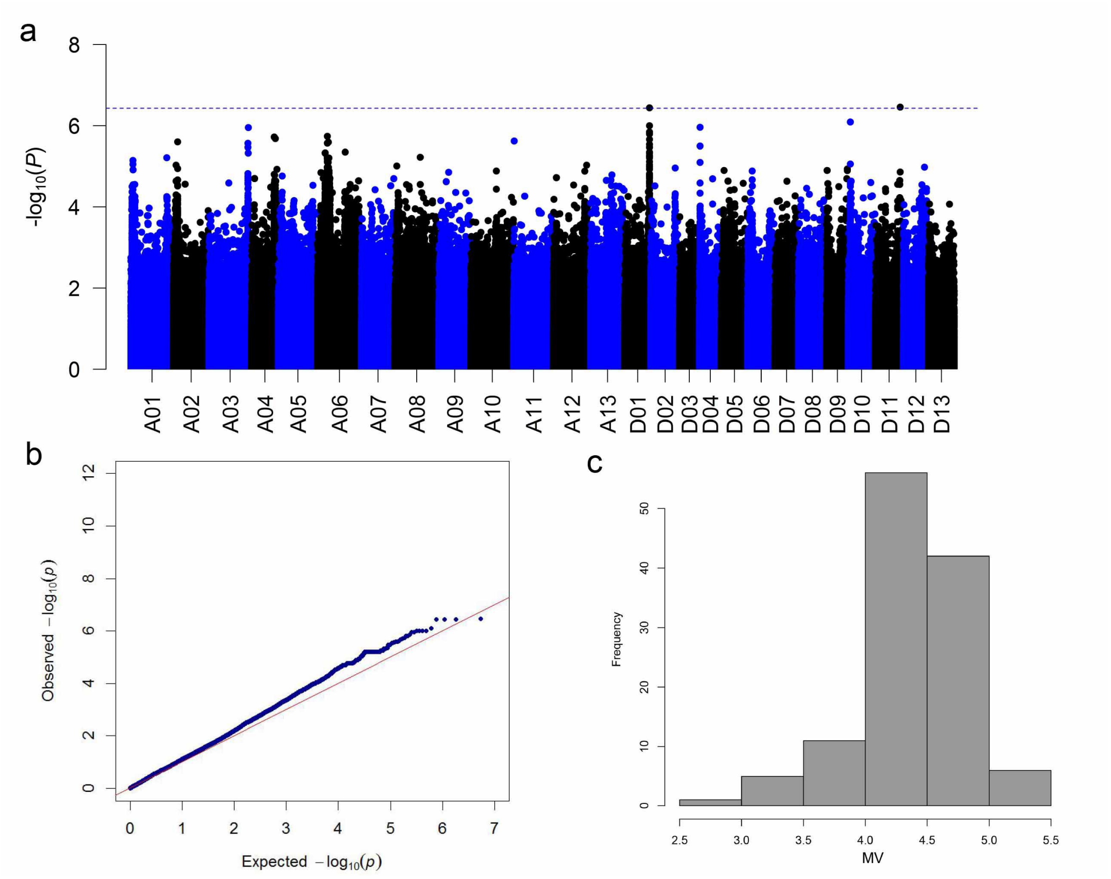

Figure S14. GWAS of the micronaire value (MV) in the BLUP data using the MLM (P+Q+K). (a) Manhattan plot of GWAS of MV. (b) Q-Q plot for MV. (c) Histogram of MV in the BLUP data.

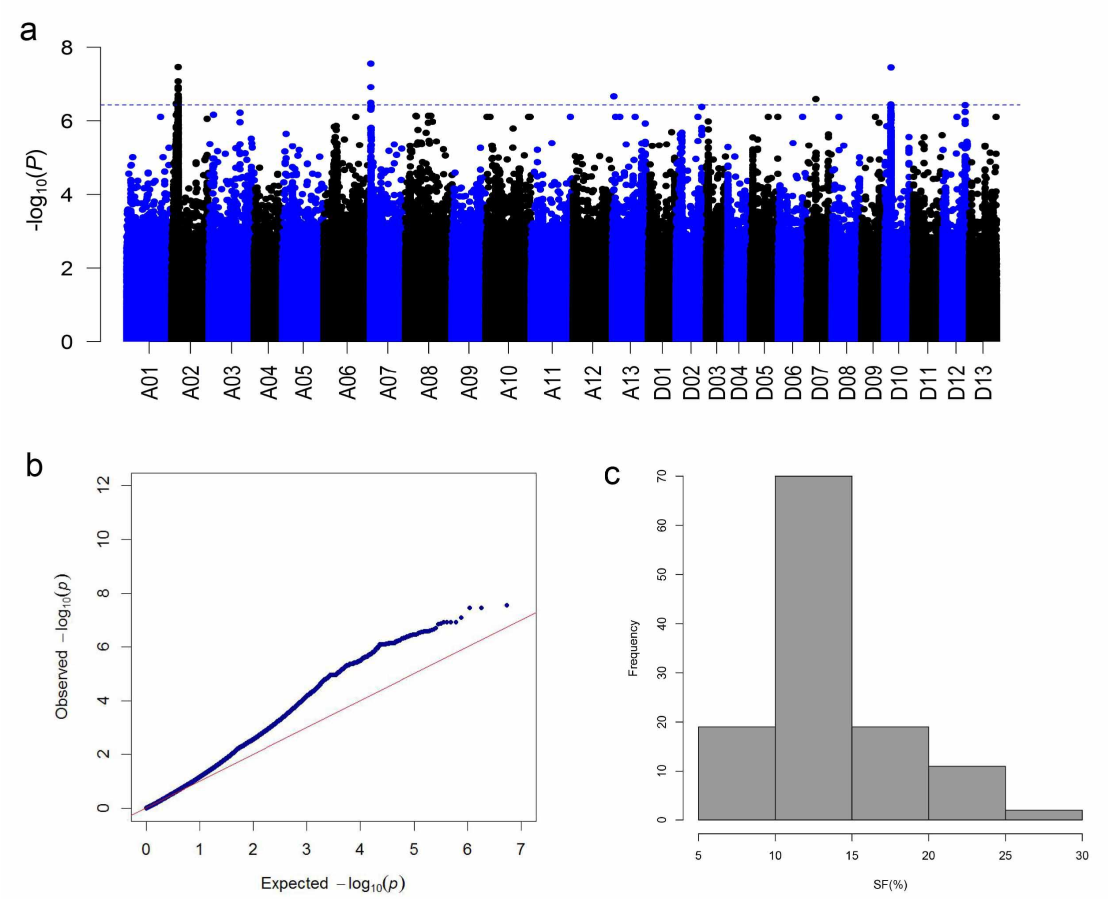

Figure S15. GWAS of the short fibre percentage (SF) in the BLUP data using the MLM (P+Q+K). (a) Manhattan plot of GWAS of SF. (b) Q-Q plot for SF. (c) Histogram of SF in the BLUP data.

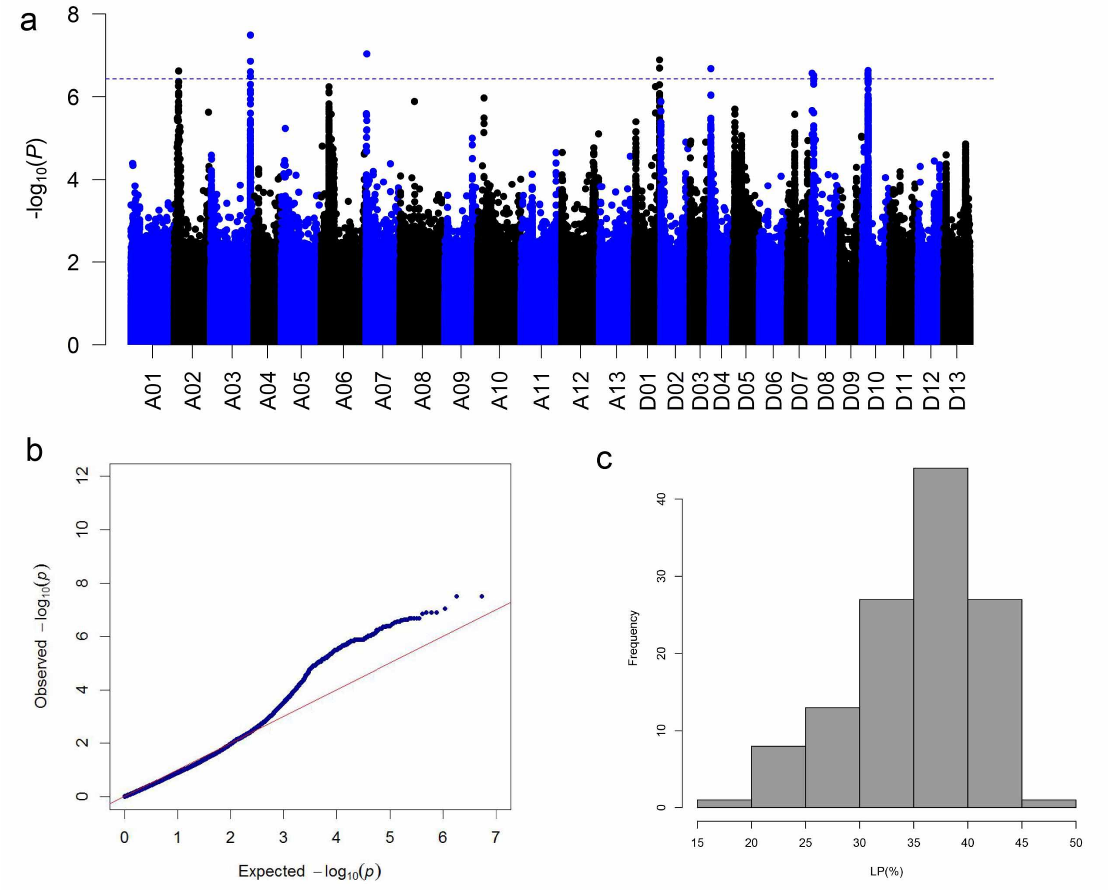

Figure S16. GWAS of the lint percentage (LP) in the BLUP data using the MLM (P+Q+K). (a) Manhattan plot of GWAS of LP. (b) Q-Q plot for LP. (c) Histogram of LP in the BLUP data.

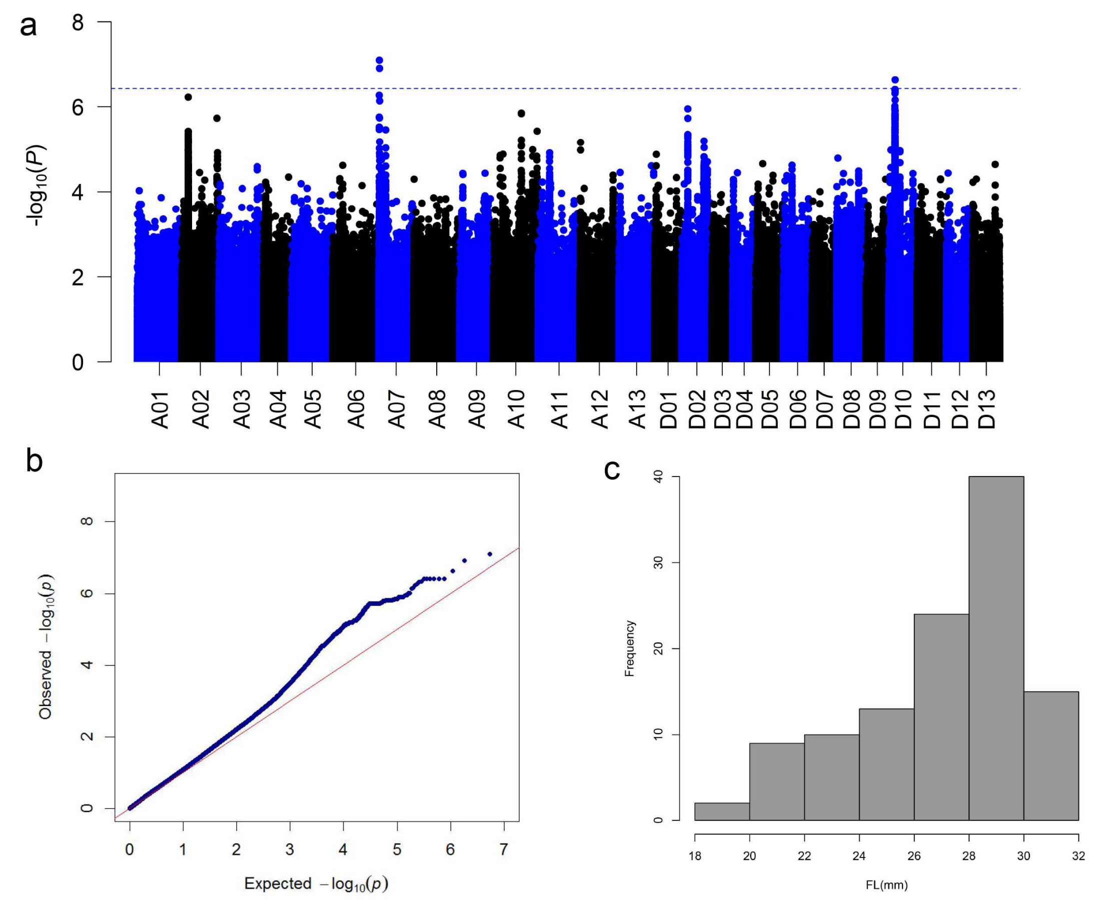

Figure S17. GWAS of the fibre length (FL) in the HG15 environment using the MLM (P+Q+K). (a) Manhattan plot of GWAS of FL. (b) Q-Q plot for FL. (c) Histogram of FL in the HG15 environment.

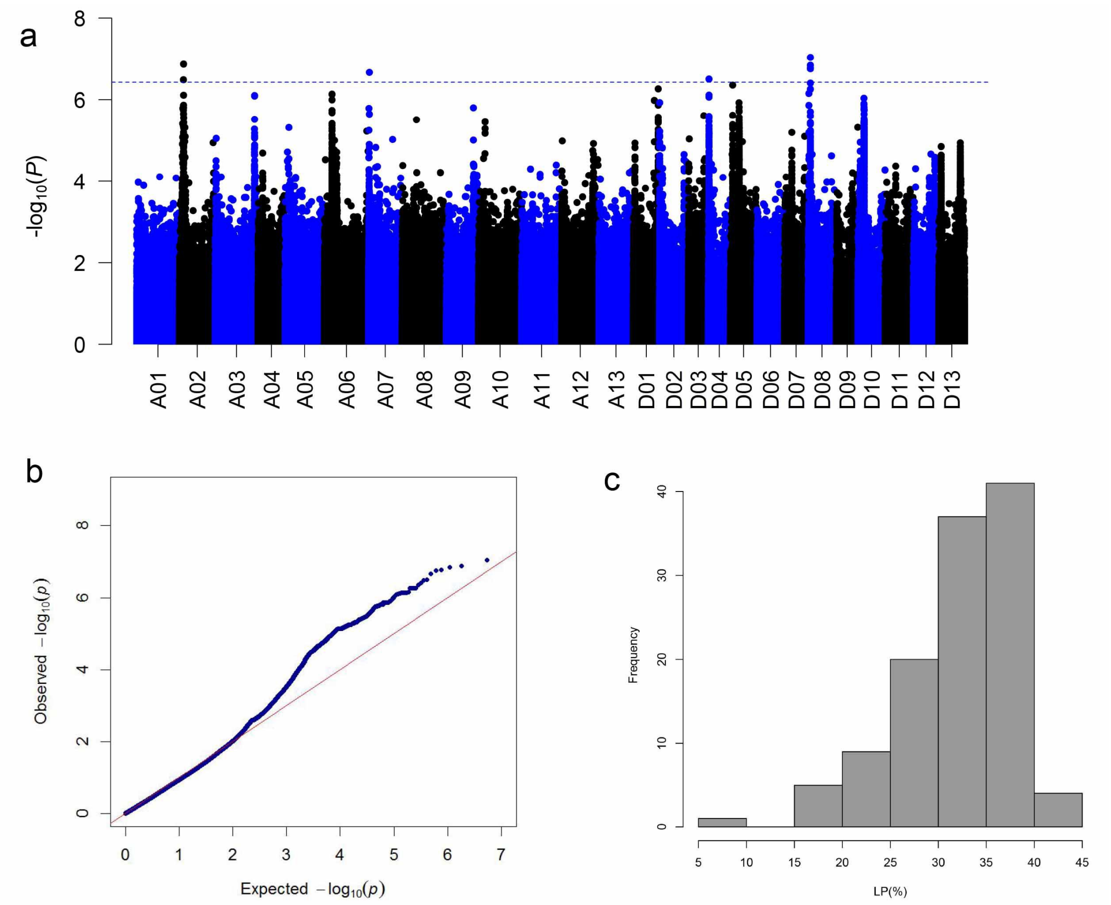

Figure S18. GWAS of the lint percentage (LP) in the HG15 environment using the MLM (P+Q+K). (a) Manhattan plot of GWAS of LP. (b) Q-Q plot for LP. (c) Histogram of LP in the HG15 environment.

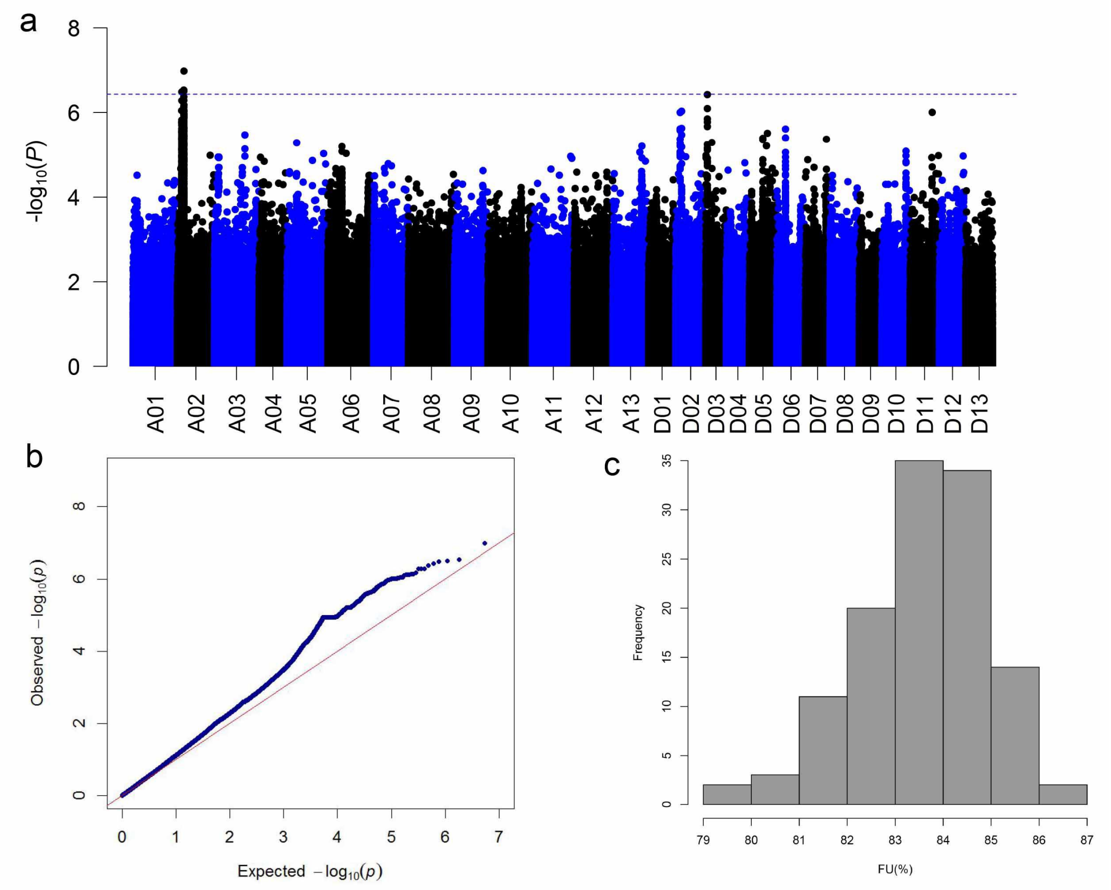

Figure S19. GWAS of the fibre unity (FU) in the XJ15 environment using the MLM (P+Q+K). (a) Manhattan plot of GWAS of FU. (b) Q-Q plot for FU. (c) Histogram of FU in the XJ15 environment.

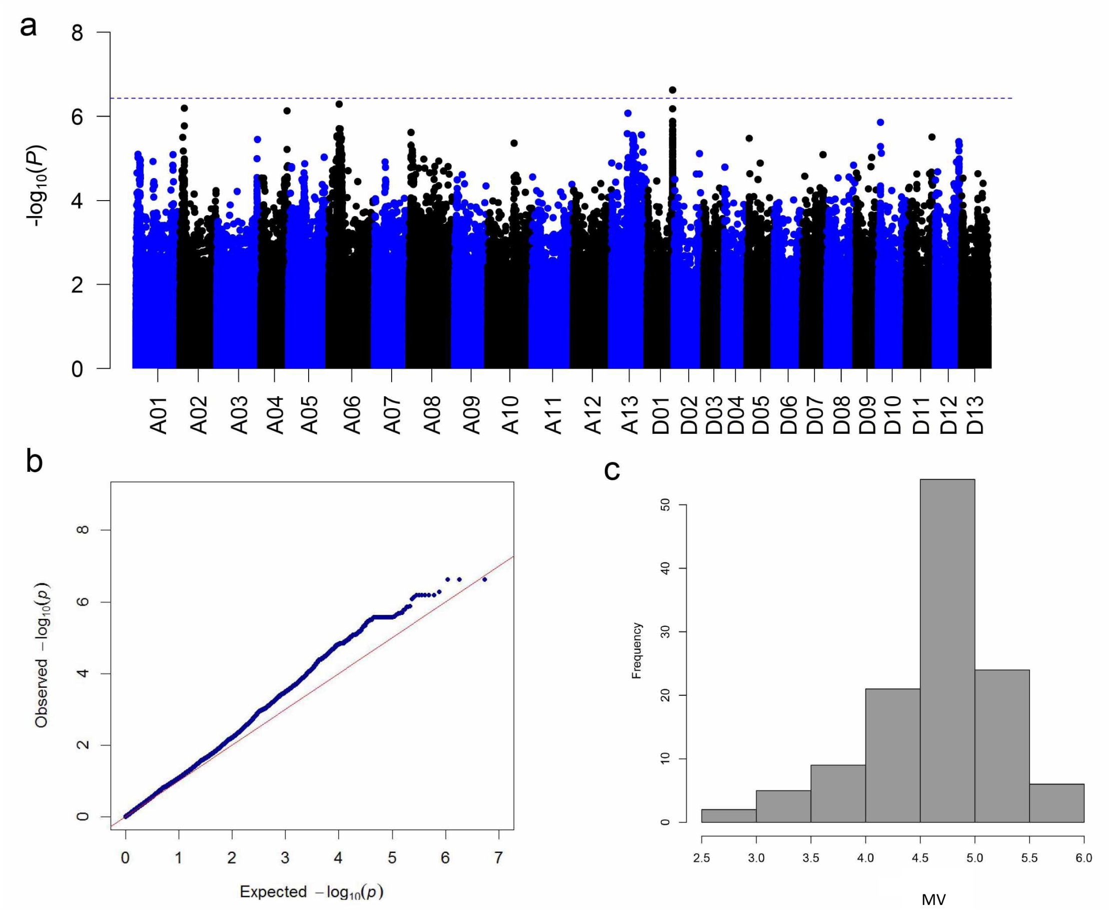

Figure S20. GWAS of the micronaire value (MV) in the XJ15 environment using the MLM (P+Q+K). (a) Manhattan plot of GWAS of MV. (b) Q-Q plot for MV. (c) Histogram of MV in the XJ15 environment.

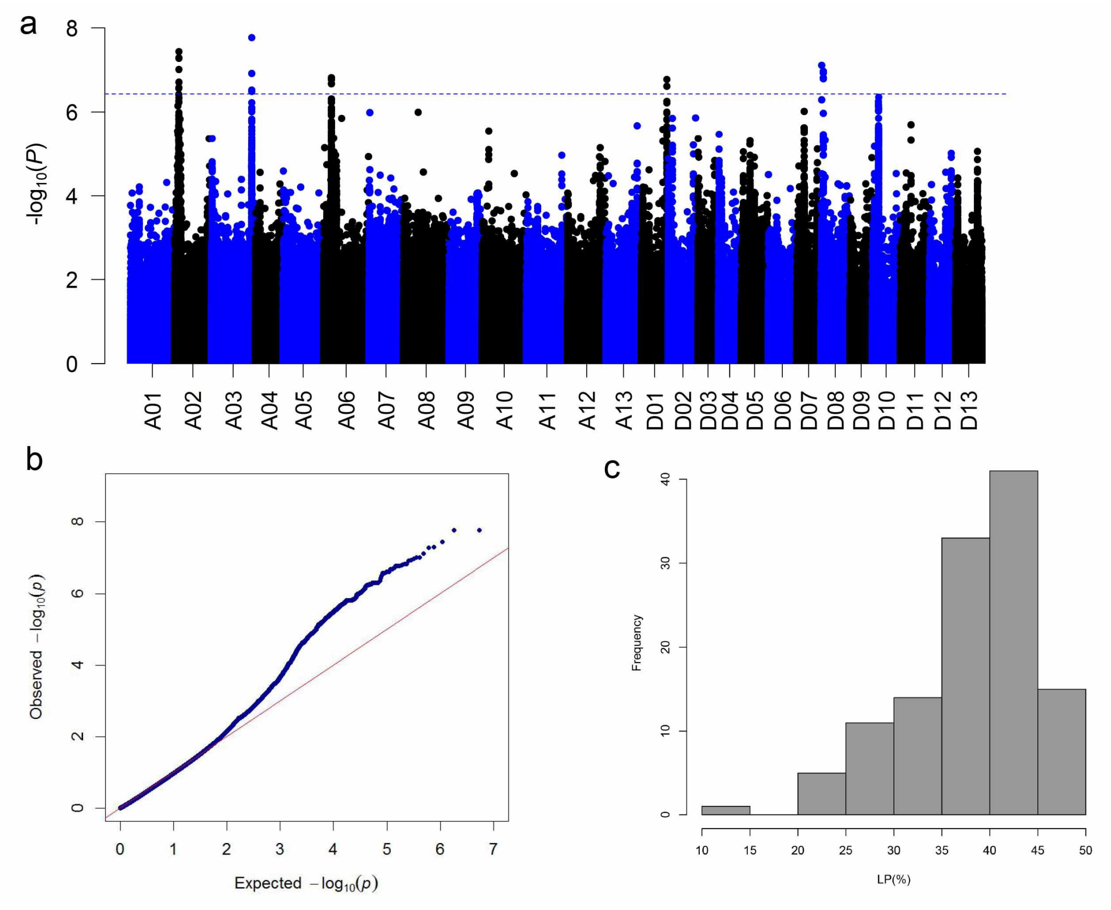

Figure S21. GWAS of the lint percentage (LP) in the XJ15 environment using the MLM (P+Q+K). (a) Manhattan plot of GWAS of LP. (b) Q-Q plot for LP. (c) Histogram of LP in the XJ15 environment.

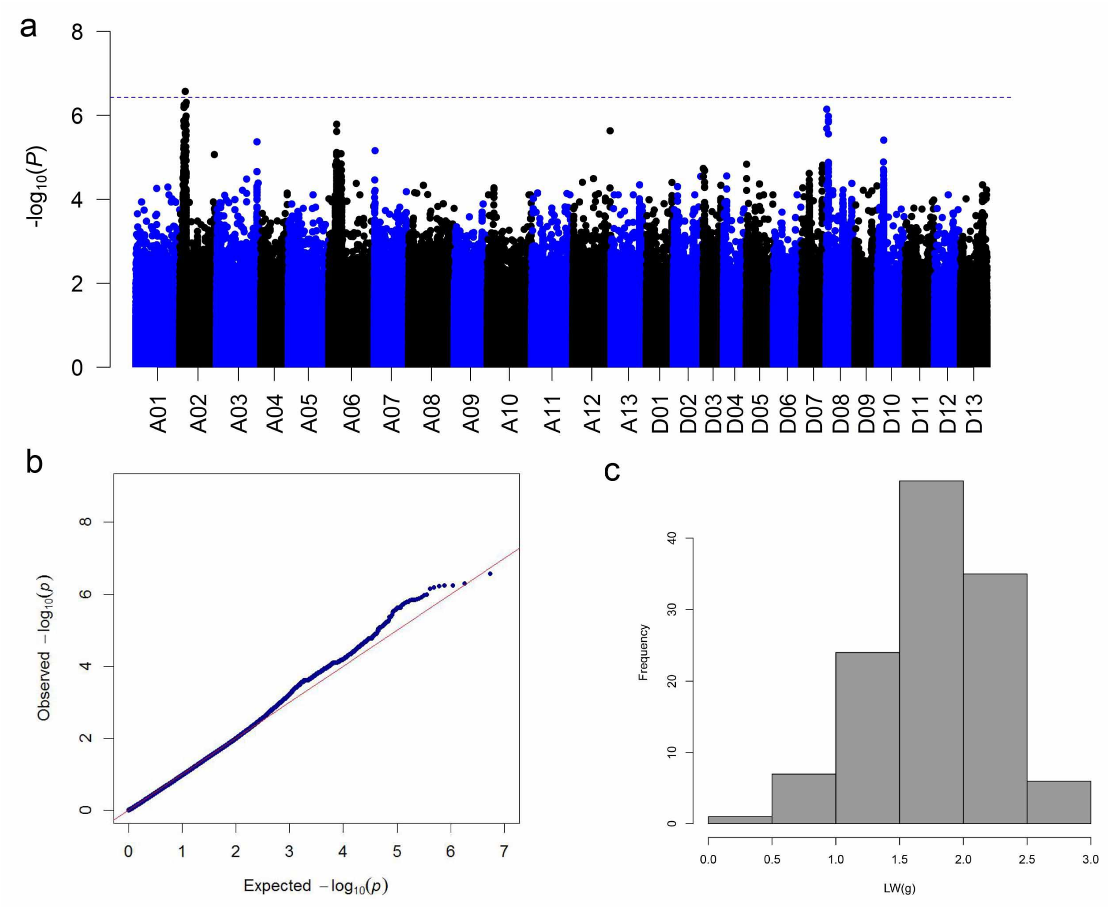

Figure S22. GWAS of the lint weight (LW) in the XJ15 environment using the MLM (P+Q+K). (a) Manhattan plot of GWAS of LW. (b) Q-Q plot for LW. (c) Histogram of LW in the XJ15 environment.

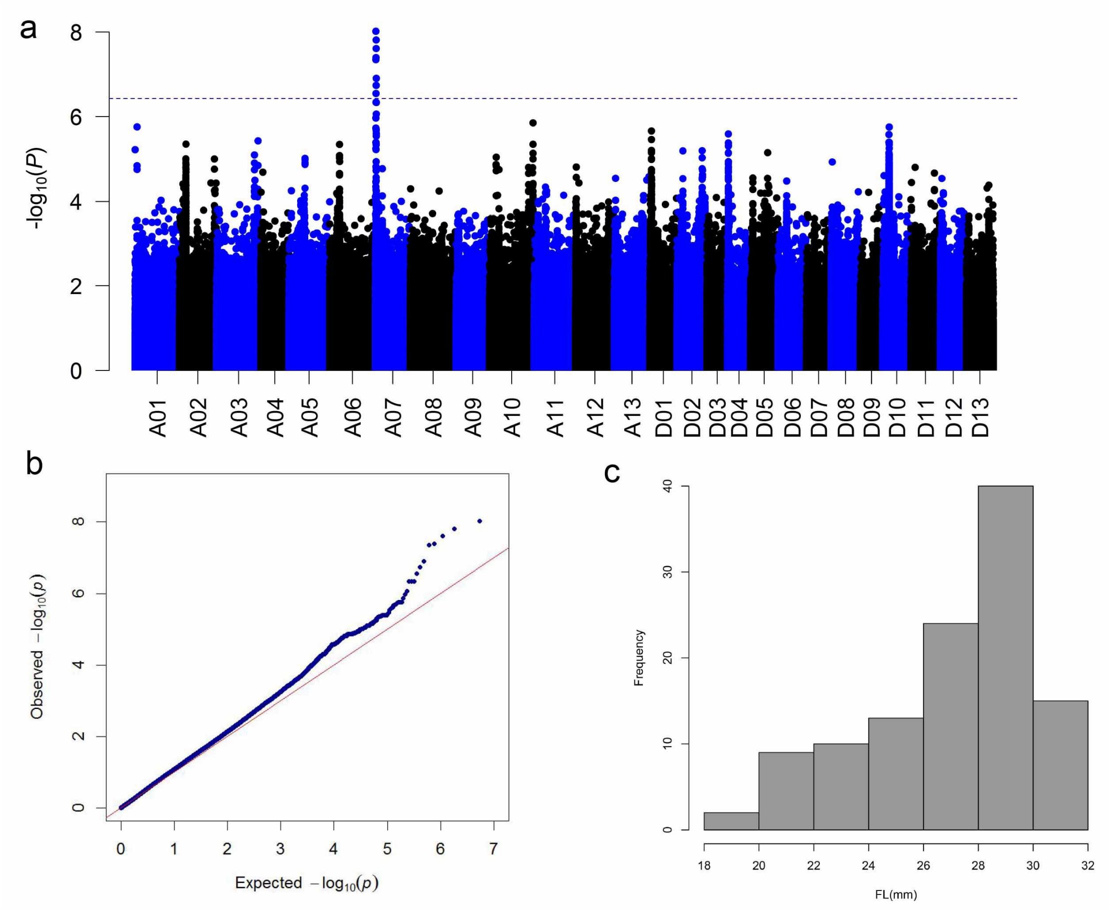

Figure S23. GWAS of the fibre length (FL) in the XJ16 environment using the MLM (P+Q+K). (a) Manhattan plot of GWAS of FL. (b) Q-Q plot for FL. (c) Histogram of FL in the XJ16 environment.

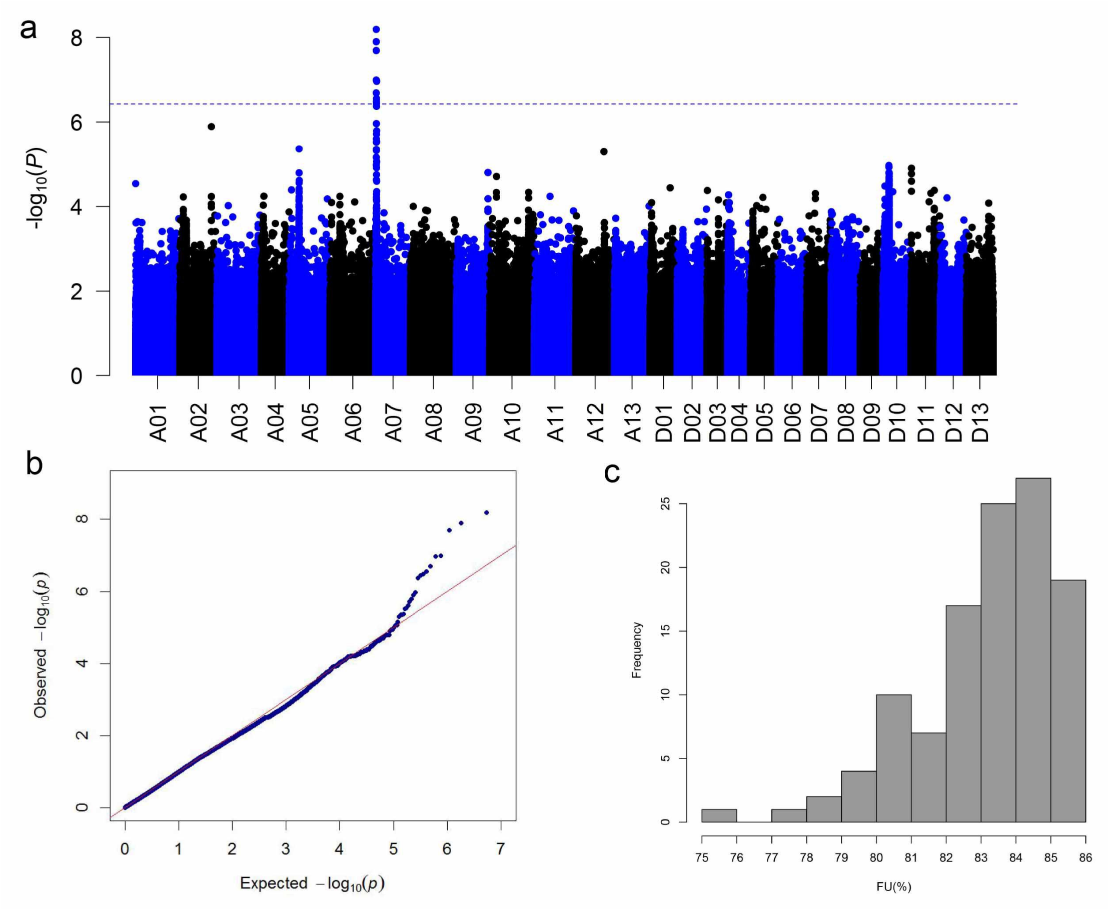

Figure S24. GWAS of the fibre unity (FU) in the XJ16 environment using the MLM (P+Q+K). (a) Manhattan plot of GWAS of FU. (b) Q-Q plot for FU. (c) Histogram of FU in the XJ16 environment.

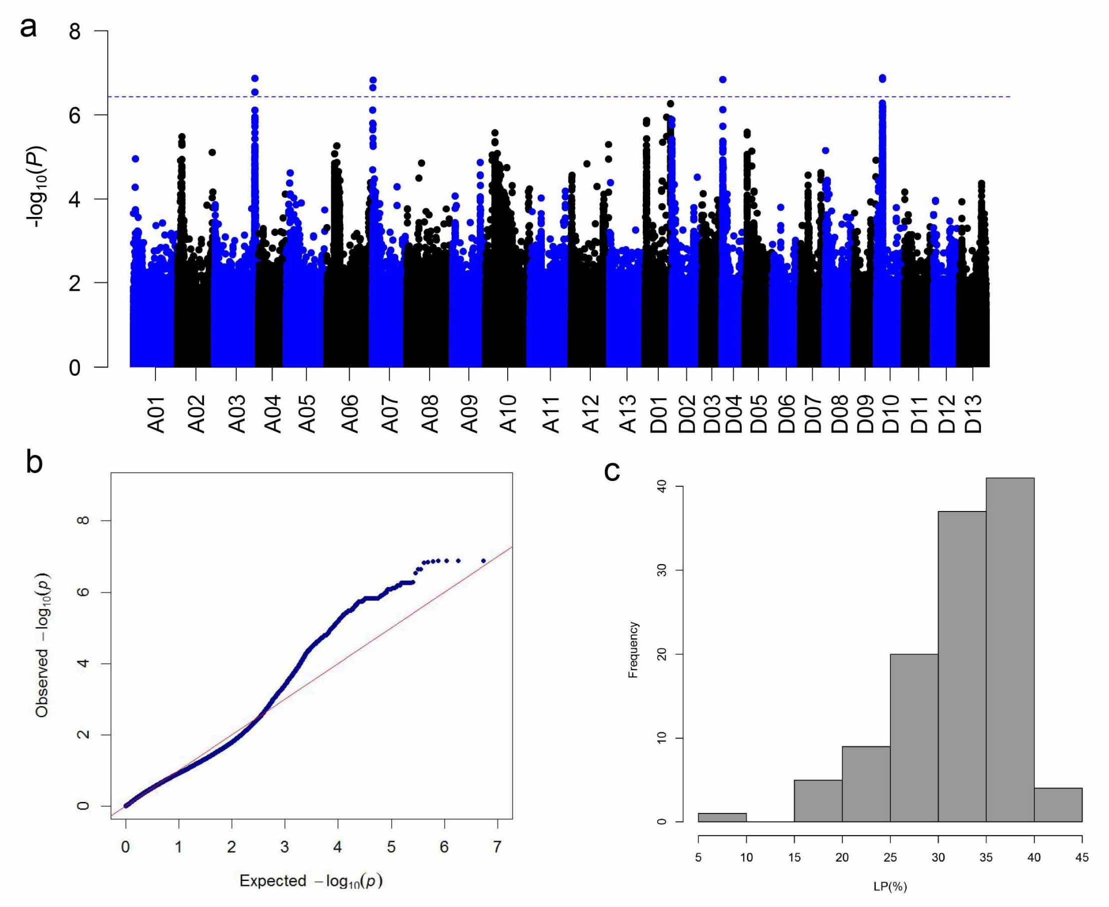

Figure S25. GWAS of the lint percentage (LP) in the XJ16 environment using the MLM (P+Q+K). (a) Manhattan plot of GWAS of LP. (b) Q-Q plot for LP. (c) Histogram of LP in the XJ16 environment.

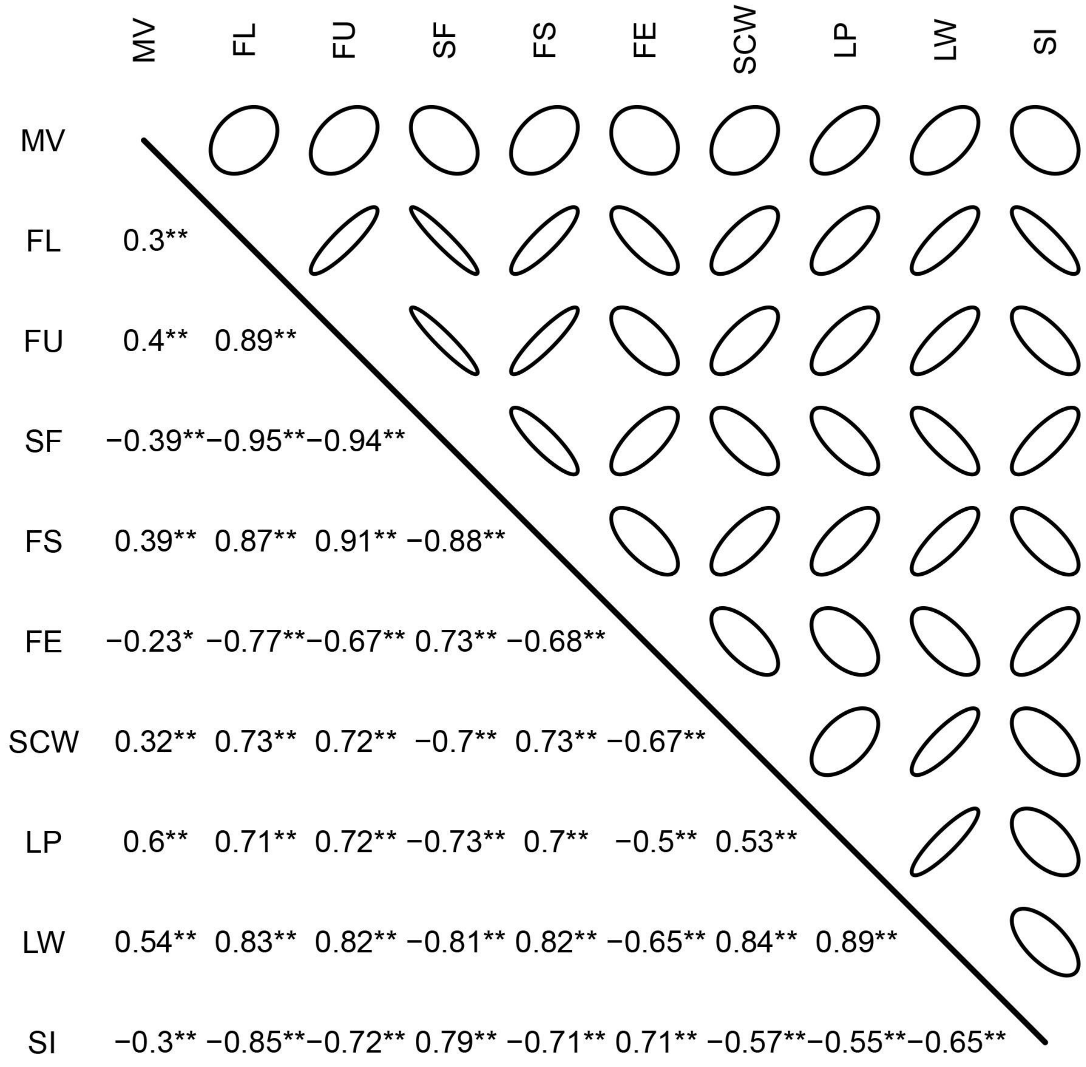

Figure S26. Correlation matrix between the shade index (SI) and nine agronomic traits in 100 brown fibre accessions. Micronaire value, MV; fibre length, FL; fibre uniformity, FU; short fibre, SF; fibre strength, FS; fibre elongation, FE; seed cotton weight, SCW; lint percentage, LP; lint weight, LW; shade index, SI. * and ** indicate the significant at $P < 0.05$ and $P < 0.01$, respectively. The shape and direction of the circles reflect the correlation between the SI and nine agronomic traits.
